# Multifaceted Defense against Antagonistic Microbes in Developing Offspring of the Parasitoid Wasp *Ampulex compressa* (Hymenoptera, Ampulicidae)

**DOI:** 10.1371/journal.pone.0098784

**Published:** 2014-06-02

**Authors:** Katharina Weiss, Christopher Parzefall, Gudrun Herzner

**Affiliations:** Evolutionary Ecology Group, Institute of Zoology, University of Regensburg, Regensburg, Germany; University of Freiburg, Germany

## Abstract

Effective antimicrobial strategies are essential adaptations of insects to protect themselves, their offspring, and their foods from microbial pathogens and decomposers. Larvae of the emerald cockroach wasp, *Ampulex compressa*, sanitize their cockroach hosts, *Periplaneta americana*, with a cocktail of nine antimicrobials comprising mainly (*R*)-(-)-mellein and micromolide. The blend of these antimicrobials has broad-spectrum antimicrobial activity. Here we explore the spatio-temporal pattern of deployment of antimicrobials during the development from egg to adult as well as their physico-chemical properties to assess how these aspects may contribute to the success of the antimicrobial strategy. Using gas chromatography/mass spectrometry (GC/MS) we show that larvae start sanitizing their food as soon as they have entered their host to feed on its tissue. Subsequently, they impregnate the cockroach carcass with antimicrobials to create a hygienic substrate for cocoon spinning inside the host. Finally, the antimicrobials are incorporated into the cocoon. The antimicrobial profiles on cockroach and wasp cocoon differed markedly. While micromolide persisted on the cockroaches until emergence of the wasps, solid-phase microextraction sampling and GC/MS analysis revealed that (*R*)-(-)-mellein vaporized from the cockroaches and accumulated in the enclosed nest. In microbial challenge assays (*R*)-(-)-mellein in the headspace of parasitized cockroaches inhibited growth of entomopathogenic and opportunistic microbes (*Serratia marcescens*, *Aspergillus sydowii*, *Metarhizium brunneum*). We conclude that, in addition to food sanitation, *A. compressa* larvae enclose themselves in two defensive walls by impregnating the cocoon and the cockroach cuticle with antimicrobials. On top of that, they use vaporous (*R*)-(-)-mellein to sanitize the nest by fumigation. This multifaceted antimicrobial defense strategy involving the spatially and temporally coordinated deployment of several antimicrobials in solution and vapor form has apparently evolved to reliably protect the larvae themselves and their food against a broad range of antagonistic microbes.

## Introduction

Owing to the persistent selection pressure exerted by detrimental microorganisms, insects have evolved diverse sophisticated antimicrobial strategies to defend themselves, their offspring, and their foods from microbial pathogens and decomposers [1], [2], [3], [4], [5], [6], [7], [8], [9], [10], [11], [12], [13]. The remedial measures include complex immune systems [14], [15], behavioral adaptations [16], [17], [18], defensive symbiosis [9], [19], [20], [21], and the deployment of chemical compounds with antimicrobial effects [1], [2], [7], [22], [23], [24]. In the latter case, the composition and specific spatio-temporal pattern of deployment of antimicrobial secretions are likely to be crucial to the success of the antimicrobial strategy [1], [23], [25], [26].

In a previous study we presented first insights into the antimicrobial defense strategy of the emerald cockroach wasp *Ampulex compressa* F. (Hymenoptera: Ampulicidae) [1]. Females of this solitary parasitoid wasp hunt and store cockroaches like the American cockroach *Periplaneta americana* (Blattaria: Blattidae) as food for their larvae [27], [28], [29], [30]. The female wasp attacks the cockroach, stings it and drags the then docile host to a nest, where it attaches an egg to the coxa of a mesothoracic leg. The female then tightly closes the nest with twigs, leaves, stones etc. and thus virtually immures the cockroach inside the nest. When the egg hatches, the larva first remains at the oviposition site on the host thorax and imbibes hemolymph through a hole in the cuticle of the still living cockroach. Subsequently, the larva moves inside the cockroach and feeds on the interior organs, causing the death of the host. Finally, the larva forms its cocoon inside the then empty cockroach carcass. Until its emergence as adult wasp the developing individual remains surrounded by the cocoon and the cockroach cuticle.

During all stages of development the wasp offspring can be afflicted by various antagonistic microbes. Their cockroach hosts, which concurrently represent food as well as microenvironment, frequently carry diverse and numerous microbes, including entomopathogenic strains [1], [31], [32], [33], [34], [35], [36]. Furthermore, opportunistic microbes, which are likely to occur in the nest environment, like e.g. mold fungi, may pose severe threats to *A. compressa* immatures, either as pathogens or as food competitors that may produce and emit harmful toxins [5], [37], [38].

To mitigate these hazards from pathogens and food competitors *A. compressa* larvae apply an antimicrobial secretion to the inside of their cockroach hosts [1]. This secretion comprises the two major components (*R*)-(-)-mellein [(*R*)-(-)-3,4-diydro-8-hydroxy-3-methylisocoumarin; „mellein“ in the following] and micromolide [(4*R*,9*Z*)-octadec-9-en-4-olide], as well as seven structurally related minor compounds. The isocoumarin mellein and the γ-lactone micromolide have been shown to be inhibitory towards a broad spectrum of bacteria, mycobacteria, fungi and a virus [1], [39], [40], [41], [42], [43], [44], [45]. Several of the minor compounds of the larval secretion, namely (*R*)-octadecan-4-olide, 7-hydroxymellein, 4-hydroxymellein, and 5-hydroxymellein [1] also seem to have broad antimicrobial activity [44], [45], [46], [47], [48]. The ultimate evolutionary and ecological function of this blend of compounds thus seems to be the protection of the larval food from an unpredictable array of competing, putrefactive microbes and of the *A. compressa* offspring themselves from pathogens.

Over evolutionary times, the strong selective pressures by microbial pathogens and food competitors presumably have not only shaped the specific combination of antimicrobials in the larval secretion, but also the way in which the antimicrobials are utilized. In the present study we thus traced the spatio-temporal pattern of deployment of the larval antimicrobials in *A. compressa* nests.

Using gas chromatography/mass spectrometry (GC/MS) we analyzed and compared the chemistry of the two protective layers surrounding *A. compressa* pupae, the parasitized cockroaches and the cocoons, separately to unravel the spatial distribution of the antimicrobials. Furthermore, we elucidated the temporal pattern of deployment of the antimicrobials on parasitized cockroaches from oviposition until emergence of the adult wasp. Owing to its volatility we found mellein in the headspace of parasitized cockroaches and hypothesized that mellein vaporizing from the cockroach has the potential to sanitize the nest space. We therefore tested for an antimicrobial effect of mellein in its vapor form against the entomopathogenic bacterium *Serratia marcescens*, the entomopathogenic fungus *Metarhizium brunneum*, and of two filamentous mold fungi newly isolated from cockroaches (*Aspergillus nomius* and *Aspergillus sydowii*). Based on our results we propose that *A. compressa* immatures mount three lines of chemical defense to deter potential pathogens and food competitors.

## Results

### Development of *A. compressa* on *P. americana*

As a prerequisite for the analyses of the spatio-temporal deployment of larval antimicrobials described below, we first documented the development of *A. compressa* on *P. americana* from oviposition to adult emergence under our laboratory conditions. The observations revealed that on day three after oviposition (3±0 days) all larvae had hatched from the eggs. During the next days the larvae grew steadily before they migrated inside of their host on average 6.7±1.5 days after oviposition. About one day later (7.9±2.1 days after oviposition) the cockroaches were dead and their abdomens appeared conspicuously swelled. Diaphanoscopy revealed the first signs of cocoons inside the cockroaches on average 9.0±2.9 days after oviposition, and the cocoons appeared to be completed within the next day (9.8±2.4 days after oviposition). The new adult wasps emerged on average 39±1.2 days after oviposition.

### Spatial distribution of the antimicrobials

In order to allow for separate chemical analysis of the host cockroach and the *A. compressa* cocoon by GC/MS, *A. compressa* larvae were transferred from their cockroach hosts to glass vials after they had eroded the host tissue but before the onset of cocoon spinning. The glass vials functioned as “ersatz hosts” in which the larvae spun their cocoons (Figure S1).

**Figure 1:**
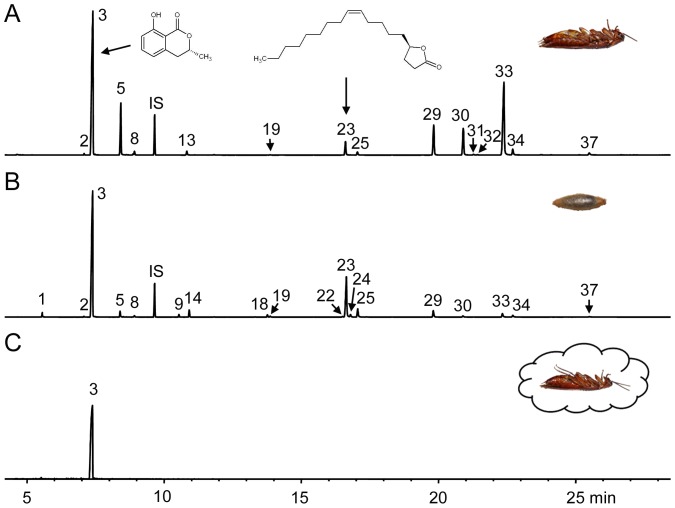
Chemical profiles of parasitized *P. americana* and *A. compressa* cocoons. Total ion chromatograms of (A) a solvent extract of a parasitized *P. americana* cockroach, (B) a solvent extract of a cocoon of *A. compressa*, and (C) a headspace sample of a parasitized *P. americana* cockroach obtained by solid-phase microextraction. Although the relative amounts of compounds in solvent extract showed a broad variation, the chromatograms can be considered typical. The peaks of minor compounds may not be visible due to the magnification used. Peak numbers correspond to the numbers in Table 1, Peak 3 =  (*R*)-(-)-mellein, Peak 23 =  micromolide. IS: internal standard.

The chemical compounds found on parasitized cockroaches and *A. compressa* cocoons are given in Table 1 and Figure 1. Cocoons consistently carried 35 substances, 21 of which were hydrocarbons. Among the thirteen more polar compounds were the isocoumarins and γ-lactones previously described from the secretion of *A. compressa* larvae [1]. Mellein was the by far most abundant component, followed by micromolide and (*R*)-octadecan-4-olide. Besides the isocoumarins and γ-lactones we additionally found four compounds (compounds 1, 2, 7, 15) which have previously not been described from *A. compressa*. The mass spectrum of compound 1 [linear retention index (LRI) 1367] had a molecular ion at *m/z* 168 (5) and fragment ions at 123 (15), 100 (26), 69 (100), and 41 (39) and matched mass spectral data reported for the acyclic monoterpene 3,7-dimethyl-2,6-octadienoic acid [49]. To confirm its identity we purchased a synthetic mixture of isomers containing (*E*)-3,7-dimethyl-2,6-octadienoic acid (geranic acid) and (*Z*)-3,7-dimethyl-2,6-octadienoic acid (nerolic acid). The synthetic compounds and the natural compound had identical mass spectra. Furthermore, the natural compound coeluted with the earlier eluting nerolic acid [49]. Therefore the identity of compound 1 could be confirmed as nerolic acid.

**Table 1:** List of chemical compounds on cockroaches and cocoons.

No.	LRI	Compound	Cocoon [µg]	Cockroach [µg]
1	1367	**Nerolic acid** *	1.5±2.2	-
2	1540	**Reacetophenone** *	0.8±1.0	4.4±2.6
3	1572	**(** ***R*** **)-(-)-Mellein**	1349.1±952	3574.0±1466
4	1671	Heptadecadiene	0.3±0.2	-
5	1687	**7-Hydroxymellein**	8.0±14.6	499.3±350.7
6	1700	Heptadecane	0.7±0.4	0.03±0.2
7	1710	**Unidentified 1**	0.4±0.8	3.0±3.0
8	1735	**4-Hydroxymellein**	8.9±9.2	53.4±25.7
9	1870	Nonadecadiene	8.3±3.6	0.03±0.1
10	1876	Nonadecene	0.3±0.2	-
11	1880	Nonadecene	0.3±0.2	-
12	1890	Nonadecene	0.2±0.1	-
13	1893	**5-Hydroxymellein**	0.2±0.3	31.7±17.9
14	1900	Nonadecane	18.6±6.3	0.4±0.5
15	1915	**Unidentified 2**	0.02±0.1	1.0±1.3
16	2000	Eicosane	0.2±0.1	-
17	2069	Heneicosadiene	1.0±0.8	-
18	2100	Heneicosane	6.8±2.3	0.1±0.2
19	2101	**(** ***R*** **)-Hexadecan-4-olide**	10.0±9.1	7.6±7.9
20	2212	**Heptadecan-4-olide**	0.8±0.9	0.8±1.1
21	2270	Tricosadiene	0.2±0.2	-
22	2281	**Octadeca-9,12-dien-4-olide**	11.2±8.5	8.5±8.3
23	2290	**Micromolide**	443.8±291.8	317.9±277.2
24	2300	Tricosane	9.0±3.2	0.9±0.7
25	2317	**(** ***R*** **)-Octadecan-4-olide**	66.5±50.6	61.9±57.3
26	2338	9-Methyl tricosane	0.5±0.4	-
27	2400	Tetracosane	0.3±0.2	0.7±0.6
28	2474	3-Methyl tetracosane	-	0.9±0.4
29	2500	Pentacosane	19.9±9.2	179.4±37.7
30	2574	3-Methyl pentacosane	4.3±2.8	177.9±40.2
31	2600	Hexacosane	0.2±0.2	5.6±2.7
32	2610	3,7-Dimethyl pentacosane	0.03±0.1	3.8±3.5
33	2674	6,9-Heptacosadiene	17.2±11.2	663.3±132.2
34	2700	Heptacosane	6.0±2.9	33.3±7.5
35	2774	3-Methyl heptacosane	-	3.0±0.8
36	2800	Octacosane	0.005±0.02	1.7±1.4
37	2900	Nonacosane	1.7±1.1	22.2±8.2
		**Sum of all larval substances**	1901±1341.1	4563.4±2219

Chemical composition of solvent extracts of parasitized *P. americana* cockroaches and *A. compressa* cocoons as well as mean amounts [µg] ± standard deviation of substances found in single extracts (*n* = 28). Compounds derived from *A. compressa* larvae (‘larval substances’) are in boldface type.

LRI =  linear retention index (calculated in relation to *n*-alkanes) on a RH-5ms+ column.

*Identified by comparing mass spectrum and retention time with a synthetic reference compound.

All other compounds were identified by comparison with data from earlier analyses [1], [53].

The mass spectrum of newly detected compound 2 (LRI 1540) exhibited a molecular ion at *m*/*z* 152 (46) and fragment ions at 137 (100), 123 (1), 109 (2), 81 (10), 69 (6), 53 (6) and 43 (4) and matched mass spectral data published for the phenolic ketone resacetophenone (2,4-dihydroxyacetophenone) [50]. The identification was confirmed by comparison with a synthetic resacetophenone sample which agreed in its mass spectral data and retention time with the natural product. The minor compounds 7 and 15 could not be identified.

Parasitized cockroaches consistently carried 28 substances, 16 of which were hydrocarbons. The twelve more polar compounds comprised the antimicrobials previously found in the larval secretion of *A. compressa* and on parasitized cockroaches [1]. The major compound was mellein, followed by 7-hydroxymellein and micromolide. Furthermore, we found resacetophenone, but not nerolic acid on parasitized cockroaches.

While cocoons already carried large amounts of larval substances, the amounts on parasitized cockroaches were even higher (Table 1; paired *t* test: *t* = −7.1, *df* = 13, *p*<0.0001). Moreover, the distribution of the two major compounds differed between cocoons and cockroach carcasses. The mean amount of mellein was significantly higher on cockroaches (Table 1; paired *t* test: *t* = −8.0, *df* = 13, *p*<0.0001), the amount of micromolide significantly higher on cocoons (Table 1; paired *t* test: *t* = 2.3, *df* = 13, *p* = 0.032). Notably, incorporating that the average cockroach surface is almost twice as large as the average cocoon surface does not change these outcomes.

**Figure 2:**
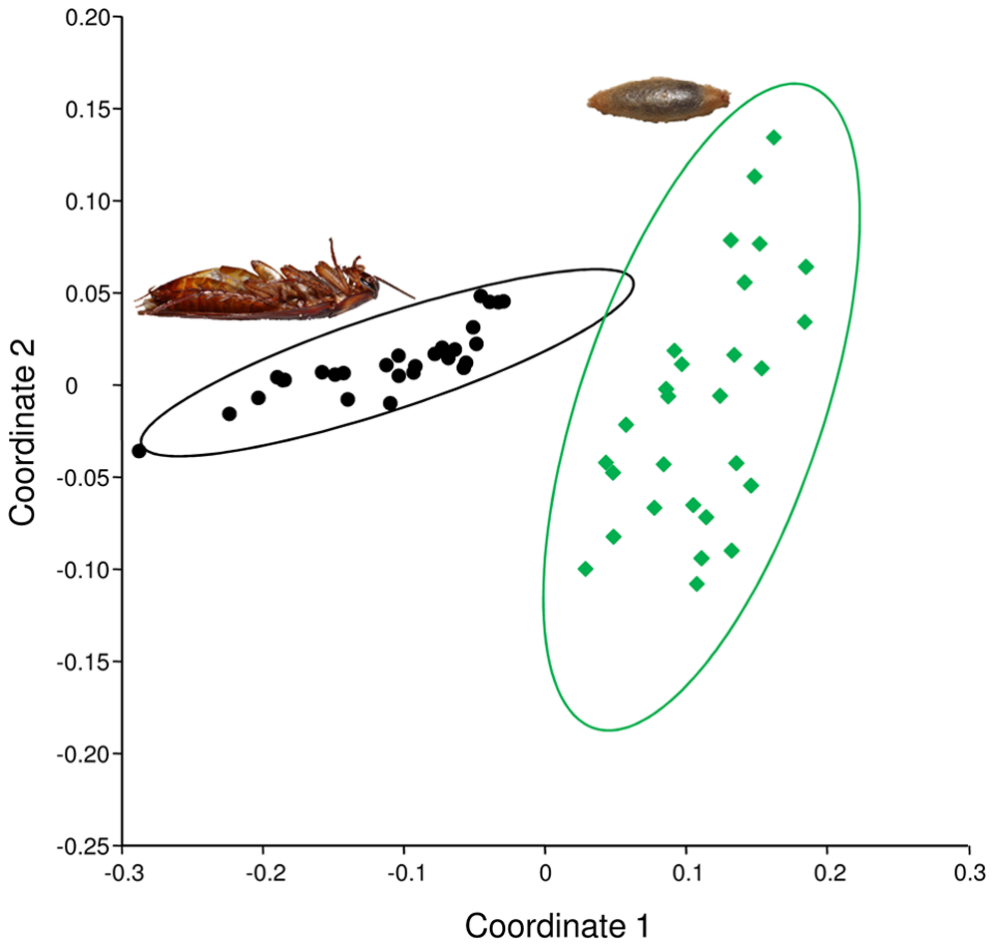
Comparison of the chemical profiles of cockroaches and cocoons. Two-dimensional MDS representation of the chemical profiles of parasitized *P. americana* cockroaches (black dots) and *A. compressa* wasp cocoons (green diamonds) (stress value: 0.03). The 95% concentration ellipses depict the regions where 95% of the points of the respective group are expected to fall.

To analyze whether the chemical profiles of antimicrobials on parasitized cockroaches and *A. compressa* cocoons differed on the whole, we compared them by multivariate statistical analyses. The two-dimensional MDS plot of the chemical profiles (Figure 2) illustrates that, despite some within-group variation, the cocoons and cockroaches can be clearly separated based on their chemical profiles. The ANOSIM yielded a highly significant difference in the chemical patterns of the two groups (*ANOSIM*: *r* = 0.9, *p* = 0.0001). The SIMPER analysis (Table S1) revealed that micromolide, which shows a higher relative abundance on cocoons, accounts for 42% of the difference between the two groups. Mellein, which shows a higher relative abundance on cockroaches, accounts for another 25%, which adds up to 67% for the two major components mellein and micromolide together.

### Temporal deployment of antimicrobials

**Figure 3:**
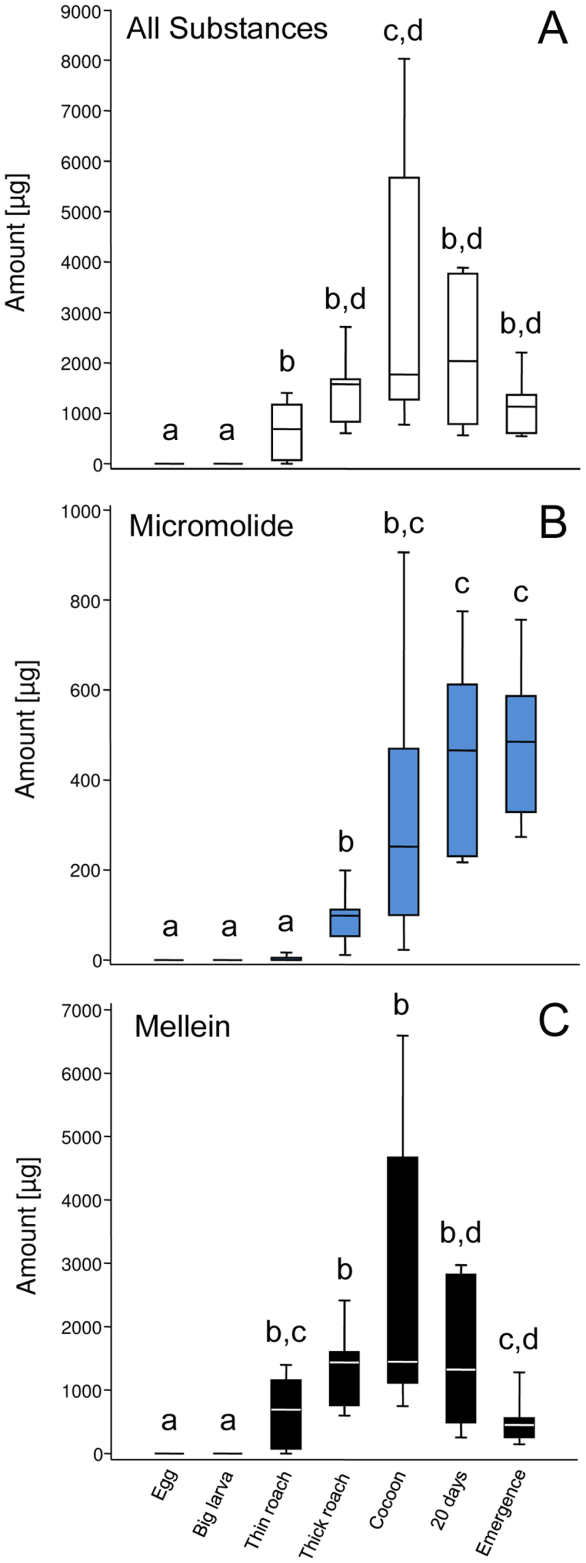
Temporal changes in the amount of larval antimicrobials in *A. compressa* nests. Median amounts of (A) all larval substances, (B) micromolide, and (C) mellein found in parasitized cockroaches of different developmental stages (*n* = 9, 10, 11, 10, 10, 9, 10; see also Figure S2). Horizontal line inside box: median, box: 25–75 percent quartiles, whiskers: minimal and maximal values. Groups that do not share at least one letter above the boxplot are significantly different. For levels of significance of the pairwise comparisons see Tables S2 and S3.

Based on the developmental data described above we analyzed the amount of substances on parasitized cockroaches of seven different developmental stages of *A. compressa* (Figure S2) to elucidate the temporal deployment of the larval antimicrobials (Figure 3A). There was an overall significant difference in the median amounts of larval antimicrobials found on parasitized cockroaches of different stages of development (Kruskal-Wallis test: *H* = 49.4, *df* = 6, *p*<0.0001). The first two groups of cockroaches, which were the cockroaches carrying eggs (“Egg”), and the cockroaches bearing big larvae on their outer cuticle (“Big larva”), showed no signs of larval antimicrobials. The antimicrobials first appeared when the larvae had migrated into the cockroaches about seven days after oviposition (“Thin roach”). Their median amount significantly increased until the cocoons were visible inside the cockroach carcasses (“Cocoon”) about ten days after oviposition and slightly decreased again until the point in time when the adult wasps eclosed from the cocoon and emerged from the cockroach about 40 days after oviposition (“Emergence”) (see Table S2 for pairwise statistical comparisons).

Micromolide could first be detected shortly after the larvae had entered their cockroach hosts, but in only three out of eleven nests of this stage. All of the cockroaches that had swelled abdomens (“Thick roach”) carried micromolide. The median amount of micromolide increased significantly during the development of the wasp larvae until the cocoon was visible. At this stage the amounts of micromolide were extremely variable. Between day 10 and day 20 (“20 days”) after oviposition the median amounts increased somewhat further to remain on a rather constant level until the adult wasps emerged (Figure 3B, overall statistical difference between the groups: Kruskal-Wallis test: *H* = 59.9, *df* = 6, *p*<0.0001; see Table S3 for pairwise comparisons).

In all samples mellein could be detected as soon as the larva had migrated into the cockroach. The amount of mellein increased with the further development of the *A. compressa* offspring until the cocoon was visible. At this stage the amounts of mellein found on individual cockroaches were considerably variable. From then on the amounts significantly decreased by more than 80% until the adult wasps emerged (Figure 3C, overall statistical difference: Kruskal-Wallis test: *H* = 51.3, *df* = 6, *p*<0.0001, see Table S3 for pairwise comparisons).

All other polar compounds first occurred in cockroaches with swelled abdomens and remained detectable until emergence of the adult wasps (except for the minor compound heptadecan-4-olide which was only detectable from the “Cocoon” stage on).

**Figure 4:**
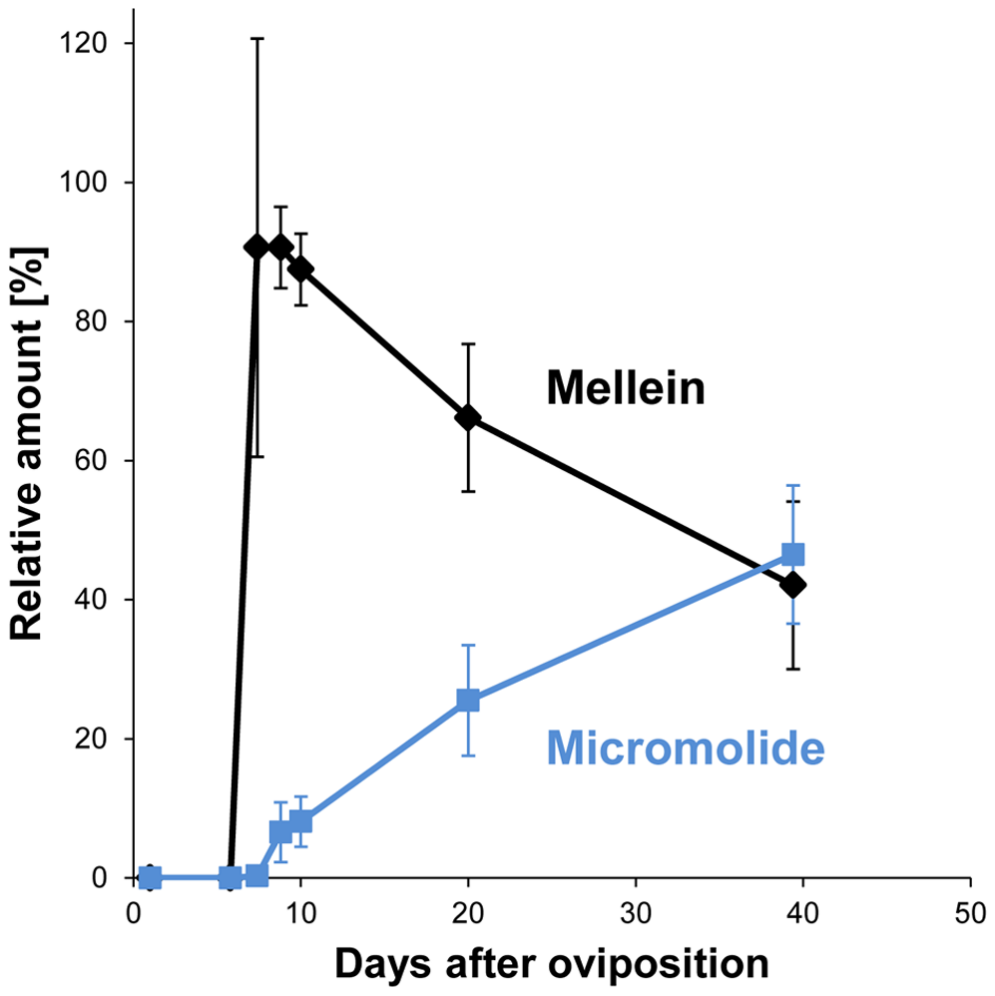
Temporal changes in relative amounts of major compounds. Relative amounts of mellein (black) and micromolide (blue) found on parasitized cockroaches during the development of *A. compressa*. Shown are means ± one standard deviation.

Notably, at the “cocoon” stage, the amounts of mellein and micromolide within one sample were significantly positively correlated (Spearman *rs* = 0.94, *p*<0.0001). The following decline of mellein and the concomitant persistence of micromolide lead to a change in their relative abundance during the development of *A. compressa* offspring (Figure 4). While mellein is the predominant compound when the larva has just entered the cockroach, its relative abundance subsequently decreases in favor of a higher proportion of micromolide. When the adult wasps emerge, mellein and micromolide are present in similar relative amounts.

### Headspace analysis of parasitized cockroaches

The relatively high volatility of mellein and the finding that its amount significantly decreased from the cocoon stage until the emergence of the adult wasps, led us to hypothesize that mellein vaporizes from the surface of the parasitized cockroaches. To test this, we collected headspace samples of six parasitized cockroaches by solid-phase microextraction (HS-SPME) and analyzed them by GC/MS. All analyzed samples contained mellein, but no other compound (Figure 1C).

### Antimicrobial challenge assays

#### Tests with parasitized cockroaches

As mellein has previously been shown to exert antimicrobial activity in broth assays [1] we conducted a series of experiments to assess the antimicrobial properties of the vaporous mellein present in the headspace of parasitized cockroaches. In these experiments agar cubes inoculated with bacteria or fungi were either incubated in the headspace of (but without direct contact to) parasitized cockroaches already containing *A. compressa* cocoons (tests) or without cockroaches (controls).

**Figure 5:**
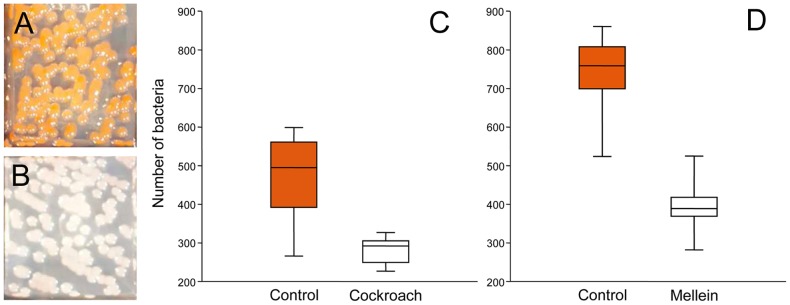
Effect of volatile antimicrobials on *S. marcescens*. (A) Reddish bacterial colonies on a control agar cube, (B) whitish bacterial colonies on an agar cube incubated in the headspace of a cockroach parasitized by *A. compressa*. (C, D) Median amount of bacteria found in (C) samples taken from control agar cubes and agar cubes incubated in the headspace of parasitized cockroaches (*n* = 10 each) and (D) samples taken from control agar cubes and agar cubes incubated in the headspace of a filter paper impregnated with synthetic mellein (*n* = 10 each). Horizontal line inside box: median, box: 25–75 percent quartiles, whiskers: minimal and maximal values.

For bacterial challenge assays we used the entomopathogen *S. marcescens*, which we had previously isolated from cockroaches and which is sensitive to mellein in liquid culture [1]. Control agar cubes incubated without cockroaches showed the conspicuous red bacterial colonies characteristic for *S. marcescens* (Figure 5A). On the agar cubes incubated in the headspace of parasitized cockroaches *S. marcescens* colonies were decolorized and had a whitish to rosy appearance (Figure 5B). The identity of the whitish bacterial colonies was confirmed as *S. marcescens* by comparing 16S rDNA sequences to those of the red colonies obtained in the controls (Text S1 and Figure S3). The mean bacterial counts in the test samples were significantly lower than in the controls (Figure 5C; Mann-Whitney *U* test: *df* = 1, *exact p* = 0.0003).

**Figure 6:**
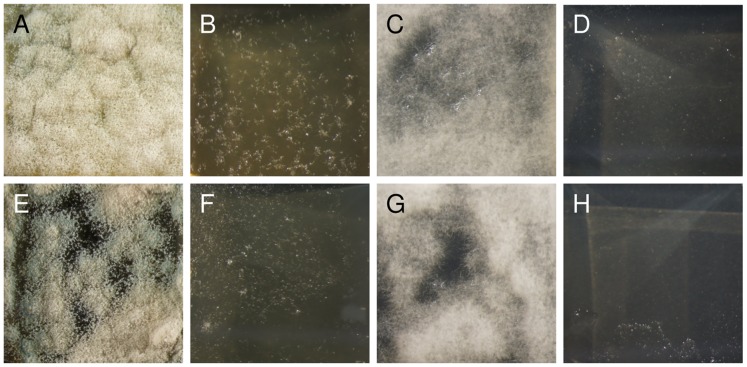
Effect of volatile compounds on *A. sydowii* and *M. brunneum*. *A. sydowii*: (A) control agar cube, (B) agar cube incubated in the headspace of a parasitized cockroach, (E) control agar cube, (F) agar cube incubated in the headspace of a filter paper impregnated with synthetic mellein. *M. brunneum*: (C) control agar cube, (D) agar cube incubated in the headspace of a parasitized cockroach, (G) control agar cube, and (H) agar cube incubated in the headspace of filter paper impregnated with synthetic mellein. All cubes were approximately 1×1×0.5 cm in size.

For fungal challenge assays we used the two filamentous fungi *Aspergillus sydowii* and *Aspergillus nomius* that we had isolated from failed *A. compressa* nests infested with these fungi (Text S1), as well as the entomopathogenic fungus *Metarhizium brunneum* as target strains. While the surfaces of control agar cubes inoculated with *A. sydowii* showed dense mycelial mats after three days of incubation (Figure 6A), the agar cubes incubated in the headspace of parasitized cockroaches showed only slight mycelial growth (Figure 6B and Table 2). A similar effect was found for *M. brunneum* that thrived well in the controls but was almost completely suppressed in the tests (Figures 6C and 6D and Table 2).

**Table 2:** Results of the antifungal challenge assays.

	*Aspergillus sydowii*	*Metarhizium brunneum*	*Aspergillus nomius*
Measure	Overgrown area [%]	Overgrown area [%]	Total mycelial area [mm^2^]
Cockroach headspace, Control vs. test	95.1±1.4 vs. 1.1±0.3#	64.3±6.8 vs. 0.1±0.06#	215.1±21.8 vs. 335.9±53.5##
Synthetic mellein, Control vs. test	71.5±1.7 vs. 0.1±0.06#	73.7±7.4 vs. 0.2±0.08#	not tested

Portions of the agar surfaces overgrown by *A. sydowii* or *M. brunneum* (median ± median absolute deviation) after incubation in the headspace of parasitized cockroaches or filter papers impregnated with synthetic mellein and the respective controls. For *A. nomius* the total areas of mycelial growth (median ± median absolute deviation) are shown (see text for further details).

*n* = 5 for all groups.

# Mann-Whitney *U* test: *df* = 1, exact *p* = 0.008.

## Mann-Whitney U test: *df* = 1, exact *p* = 0.02.

**Figure 7:**
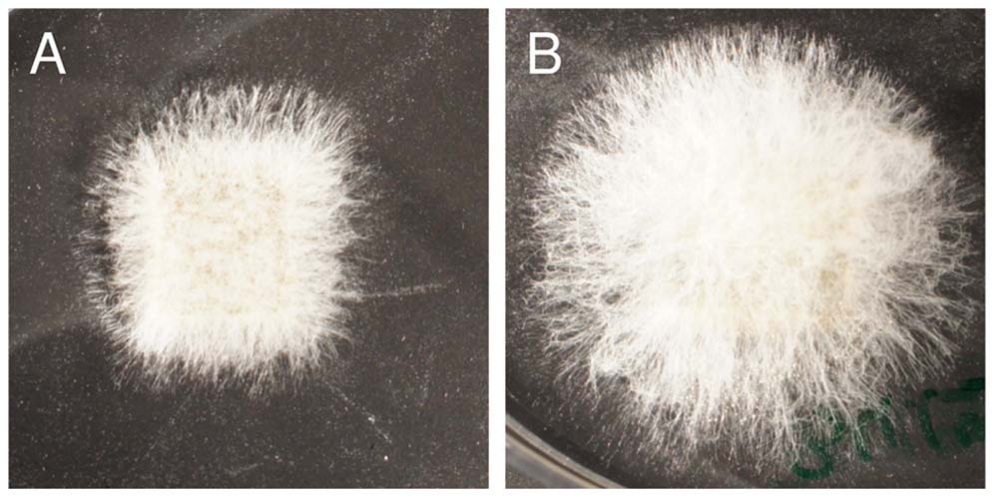
Effect of volatile compounds on *A. nomius*. (A) Control agar cube and (B) agar cube incubated in the headspace of a parasitized cockroach (agar cubes were approximately 1×1×0.5 cm in size; magnifications of photographs are equal).

The surfaces of all cubes inoculated with *A. nomius* were completely overgrown with mycelial mats after three days, irrespective of the presence of parasitized cockroaches during incubation. The hyphae on cubes of the test group appeared longer, however, and the area of mycelial growth was significantly larger in the test group as compared to the control group (Figure 7 and Table 2).

#### Tests with synthetic mellein

As mellein was the only detectable compound in the headspace of parasitized cockroaches, the results of the microbial challenge assays suggest that mellein inhibits the growth of the bacterium *S. marcescens* and the two fungi *A. sydowii* and *M. brunneum* also in its vapor form. To confirm this, we repeated the challenge assays for these microbes, but this time used filter papers impregnated with synthetic mellein instead of parasitized cockroaches. The median peak areas of mellein obtained from the headspace of these filter papers by SPME sampling and GC/MS analysis (*n* = 10; median ± median absolute deviation: 2.53×10^9^±2.63×10^8^) were similar to those obtained from the headspace of parasitized cockroaches (*n* = 10; 1.99×10^9^±3.42×10^8^) (Mann-Whitney *U* test: *df* = 1, exact *p* = 0.09).

The control agar cubes inoculated with *S. marcescens* and incubated with filter papers without mellein carried red bacterial colonies. The incubation of agar cubes with filter papers carrying synthetic mellein resulted in whitish bacterial colonies. The mean bacterial count in the test samples was significantly lower than in the controls (Figure 5D; Mann-Whitney *U* test: *df* = 1, *exact p*<0.0001).

Compared to controls (Figure 6E), the growth of *A. sydowii* was significantly reduced in the presence of filter papers impregnated with synthetic mellein (Figure 6F and Table 2). Likewise, *M. brunneum* grew well in controls (Figure 6G), but was significantly impaired in the presence of mellein-loaden filter papers (Figure 6H and Table 2).

## Discussion

By tracing the spatio-temporal pattern of deployment of larval antimicrobials, we found strong evidence that developing *A. compressa* offspring mount a multifaceted antimicrobial defense strategy to reliably and persistently protect themselves against antagonistic microbes. During their development wasp offspring may be continuously challenged by pathogenic and competing bacteria and fungi.

The first danger certainly emerges from their host cockroaches themselves, as these frequently carry various detrimental microbes [1], [31], [32], [33], [34], [35], [36], [51]. *A. compressa* larvae may thus be forced to compete with fast growing bacteria and fungi that degrade their food and may produce harmful toxins (see e.g. [5], [37], [38]). In this study we detected the antimicrobial isocoumarins and γ-lactones as soon as the larvae had migrated into the cockroaches to feed inside their hosts (Figure 3). Larvae seem to sanitize the host tissue to prevent decomposition of their sole food source as well as infection by food borne microbes [1]. By the time the cockroaches died (between the “thin roach” and the “thick roach” stage, on average eight days after oviposition), considerable amounts of antimicrobials had already accumulated in the host (Figure 3). The concentration of mellein found at that time would, for example, be sufficient to effectively impair growth of *S. marcescens* (the concentrations were 1.44 to 3.31 times the IC_50_, i.e. the concentration that is required to inhibit 50% of microbial growth, of mellein against *S. marcescens*, as estimated in [1], see also Figure S4). Such a food hygienic behavior has also been described for larvae of the ichneumonid wasp *Pimpla turionellae* [52]. In the European beewolf *Philanthus triangulum*, a solitary digger wasp [8], [53], and burying beetles of the genus *Nicrophorus* [5], [54], [55] the larval food is preserved by the parents.

An especially critical stage of development is the period of cocoon spinning. The larva has to take care that no detrimental microbes end up inside the cocoon, where they could harm or kill the larva. In the lesser spruce sawfly e.g. mortality during the cocoon stage could be ascribed to infection of the mature larvae prior to cocoon formation [56]. In *A. compressa* the highest amounts of antimicrobials were in fact found around the time of cocoon spinning (Figure 3). It seems that the larvae thoroughly disinfect the inside of the cockroach by virtually soaking it with their antimicrobial secretion to create a germfree environment for cocoon spinning.

After successful cocoon formation *A. compressa* progeny must protect themselves against opportunistic microbes that may attack from the nest surroundings during the pupal phase and metamorphosis inside the cocoon. The prepupae and pupae are generally very vulnerable stages of an insect's life cycle as they are largely sedentary, cannot display hygienic behaviors, and have mostly unsclerotized and unmelanized integuments [57]. Various insect taxa have evolved pupal cocoons that provide an important physical barrier to microbial pathogens during this period of heightened risk [3], [56], [58]. As the physical barrier alone may not be sufficient to grant survival [3], [58], insect larvae may be forced to integrate antimicrobial compounds into their cocoons to enhance protection against e.g. aggressive fungi [9], [25], [26].

Our results show that *A. compressa* larvae impregnate their cocoons with antimicrobials during cocoon spinning (Figure 1B and Table 1). The cocoons carry all the antimicrobials previously described from the larval secretion [1]. In addition to these known antimicrobials we were able to characterize two new compounds. The first, nerolic acid, was present only on cocoons, but not on cockroach carcasses. Interestingly, a mixture of nerolic acid and geranic acid has been shown to inhibit the growth of the fungus *Ascosphaera apis*, the causative agent of chalkbrood in honeybees [59]. The second compound, resacetophenone, was present on both parasitized cockroaches and cocoons. Resacetophenone isolated from a freshwater fungus was shown to have antibacterial activity [60]. Notably, both nerolic acid and resacetophenone have previously been found in conjunction with mellein in ants [49], [61]. Owing to their antimicrobial potential, the two newly characterized compounds may contribute to the protection of *A. compressa* immatures against detrimental microbes.

While both shells surrounding *A. compressa* immatures, the cocoon and the cockroach cuticle, carry antimicrobials, their chemical profiles differ markedly (Table 1 and Figure 1). The absolute and relative abundance of the major compound mellein is higher on cockroaches (Table 1), indicating that the larval secretion contains higher proportions of mellein during food preservation and cockroach sanitation. In contrast, the absolute and relative abundance of micromolide is higher on cocoons (Table 1), which points to a higher proportion of micromolide in the antimicrobial blend incorporated into the cocoon. Notably, the cockroach carcasses bear significantly higher amounts of antimicrobials than cocoons (Table 1).

As the association with the cockroach host extends over a prolonged period of time, and the cockroach carcass and cocoon must provide shelter until the adult wasp emerges, the stability of antimicrobials may be crucial. Cocoons of the European beewolf *P. triangulum* for example are protected against antagonistic microorganisms by a cocktail of antibiotics that are produced by the bacterial symbionts of the wasp [9], [25], [26]. Even though their production is confined to a relatively short period after cocoon spinning, the antibiotics present on the surface of *P. triangulum* cocoons have been shown to remain stable and supposedly protect the wasp offspring until adult emergence [26].

On parasitized *P. americana*, the persistence of the two major compounds, once they had reached their highest levels during cocoon formation, differed markedly (Figures 3 and 4). The amounts of micromolide remained rather constant until the emergence of the adult wasps. In contrast, the average amount of mellein considerably decreased after cocoon completion to about 50% 20 days after oviposition and even further to 18% until the emergence of the adult wasps.

Our headspace analyses (Figure 1C) suggest that mellein diffuses through the cockroach cuticle, vaporizes from the cockroach surface, disperses and accumulates within the confined space of the tightly closed nest and engulfs the parasitized cockroach. Previous work has shown that mellein inhibits the growth of the entomopathogenic bacterium *S. marcescens* in broth microdilution assays [1] and that it has antifungal activity [41], [42], [43], [62]. We thus hypothesized that the vaporous mellein may curtail the growth of microbes like e.g. opportunistic fungi in the surrounding nest space and may thus lower the risk of microbial attacks on the cockroaches. In fact, the growth of *S. marcescens* was reduced by 40% in the headspace of parasitized cockroaches (Figure 5C). Furthermore, the colonies that grew in the presence of the cockroaches did apparently not produce the red pigment prodigiosin (Figure 5B), which is responsible for the characteristic red coloration of many *S. marcescens* strains under normal conditions (Figure 5A) [63]. This effect of inhibition of prodigiosin synthesis in *S. marcescens* has also been observed for several antibiotics [64] and the plant-derived coumarin umbelliprenin [65].

The growth of the fungi *A. sydowii* and *M. brunneum* was almost completely suppressed in the headspace of parasitized cockroaches (Figures 6A–D). *A. nomius*, however, seemed to produce even longer hyphae in the presence of parasitized cockroaches (Figure 7). Whether this reflects a growth-promoting effect or some kind of abnormal development of the fungus is not clear. In a study on the antimicrobial activity of the oral secretion of spruce beetles, *Dendroctonus rufipennis*, *A. nomius* was the only fungus that showed no significant inhibition zones, but merely altered colony morphology [66]. In any case, growth of fungi is significantly affected in the headspace of parasitized cockroaches.

Synthetic mellein in its vapor form, in concentrations as those found in the headspace of parasitized cockroaches, likewise inhibited the growth of the bacterial and fungal target strains (Figures 5D and 6E–H). Our results hence provide strong evidence that mellein vaporizing from the cockroaches' cuticles exerts strong antimicrobial effects. The enclosed microenvironment of the nest makes fumigation with an antimicrobial volatile compound like mellein a possible defense strategy. Its volatility allows mellein to permeate the whole nest space and to affect microbes at some distance from the cockroach as well as those hidden in otherwise inaccessible sites of the nest. The vaporous mellein surrounding the parasitized cockroach thus provides a first line of defense for developing *A. compressa* against antagonistic microbes attacking from the nest environment.

It is important to note that the cockroach and cocoon are not rendered defenseless by the vaporization of mellein. Firstly, micromolide is constantly present on the cockroach and cocoon from the cocoon stage on until adult emergence. Secondly, because water evaporates from the cockroach and cocoon rather rapidly, the actual concentration of mellein in the system, which determines its antimicrobial potential, still increases until the emergence of the wasps (Figure S4; [1]).

A conclusive example of nest sanitation by fumigants has, to our knowledge, not been described for any insect so far. There are, however, some studies dealing with the possible role of volatile organic compounds as antimicrobials in insects [6], [7], [24], [67], [68], [69], [70], [71], [72], [73]. The volatiles naphthalene and fenchone that are present in the nest of *Coptotermes formosanus* termites have been hypothesized to act as fumigants in the closed nest to inhibit microorganisms [67], [68], [69], [70]. However, the amounts of these substances found in nest material are much lower than those that showed antifungal activity *in vitro* [69], [70]. Analogous results were obtained for antimicrobial benzoquinones emitted by earwigs [24]. Furthermore, chemical compounds isolated from glands of lower and higher termites have fungistatic activity not only by direct contact but also when conidia are incubated with their vapor [71], [72]. Although not directly shown, the volatile components may impregnate the nest material and render it unsuitable for fungal growth. Finally, larvae of the brassy willow leaf beetle *Phratora vitellinae* emit salicylaldehyde as a gaseous disinfectant to combat bacteria and fungi in their microenvironment [7]. Other leaf beetle genera and a ladybird beetle have also been proposed to emit volatile antimicrobials for disinfection of their microenvironment [6], [73]. However, these beetle larvae develop on leaves and not in confined nests.

Interestingly, fumigation and biofumigation are also used in human food industry to improve the microbial quality and safety of foodstuffs. In fact, the application of (preferably naturally derived) volatile antimicrobials is the most widely used and effective preservation technique to prevent post-harvest deterioration of fresh fruits and vegetables [74], [75].

## Conclusion

*A. compressa* larvae have evolved an intricate and intriguing defense strategy involving several antimicrobials with combined activity against a wide range of different microbes [1], [39], [40], [41], [42], [43], [44], [45], [46], [47], [48], [62]. Taken together, our results reveal clear spatial and temporal patterns in the deployment of the antimicrobials that seem to act jointly over a prolonged period of time to grant protection from pathogenic and competing microbes.

Inside their cockroach hosts, the larvae first sanitize their food and their immediate microenvironment from microbes that are present on the cockroaches. By doing so, they concurrently create a hygienic substrate for cocoon formation. During cocoon formation antimicrobials are also incorporated into the cocoon walls. In addition to the antimicrobial-soaked cockroach cuticle the cocoon hence provides a further protective shell against opportunistic microbes from the nest environment. The antimicrobials on the two defensive shields provide shelter until the adult wasps emerge.

A fraction of the mellein used for host sanitation penetrates the cockroach cuticle and vaporizes into the nest space, where it suppresses the proliferation of opportunistic microorganisms and, thus, provides the front line of defense. Mellein thus seems to have a triple function as food preservative during feeding, antimicrobial cover on the cockroach shell and cocoon, and fumigant in the nest space. Owing to its persistence, micromolide reliably protects the cockroach cuticle and especially the cocoon. All the components of nest and host sanitation may not only be crucial to prevent fungi from infesting the wasp immature inside the host cockroach, but also as a prophylactic strategy to minimize the risk of infection of the freshly emerged adult at eclosion from the cocoon, cockroach shell and nest. It is conceivable that the early developmental stages of *A. compressa*, i.e. the eggs and early larval instars, must likewise be protected against detrimental microbes. If and how these early stages mitigate disease risks remains to be investigated.

In order to infect *A. compressa* immatures inside the cocoons microbes would have to overcome three lines of defense: the nest space surrounding the host, the host cuticle, and the cocoon wall, each enriched with antimicrobials. This strategy is akin to the defensive fortification of a castle comprising an outer and an inner wall (cockroach cuticle and cocoon) surrounded by a moat (vaporous mellein). The three lines of defense of *A. compressa*, involving a spatially and temporally coordinated deployment of several antimicrobials in solution and in vapor form, represent an elaborate and multifaceted “better safe than sorry”-strategy that has apparently evolved to match the need for a dependable and enduring protection against an unpredictable spectrum of detrimental microbes.

## Materials and Methods

### Insects

Parasitized *P. americana* and *A. compressa* cocoons were obtained from laboratory populations that have been kept at the University of Regensburg for several years (for details see [1]). The average head capsule width and weight at emergence of *A. compressa* that develop on *P. americana* (at 27°C) are 3.3 mm and 100 mg for males and 4.2 mm and 140 mg for females (own unpublished data).

### Development of *A. compressa* on *P. americana*

*A. compressa* nests incubated in a conditioning cabinet at 27°C in the dark were monitored every 24 hrs and the state of the cockroach (*P. americana* males only) and developing *A. compressa* were documented. When the larvae had migrated inside the cockroaches and were no longer visible from the outside, diaphanoscopy (transillumination) of the cockroach using a cold light source with a fiber-optic light guide was used to monitor the state of the cockroach and developing wasp larva. Values given are means ± standard deviation.

### Identification of chemical compounds

Identification of chemical compounds was accomplished by use of gas chromatography/mass spectrometry (GC/MS). Coupled GC/MS analysis was performed with an Agilent 6890N Series gas chromatograph (Agilent Technologies) coupled to an Agilent 5973 inert mass selective detector. The GC was equipped with a nonpolar RH-5ms+ fused silica capillary column (30-m×0.25-mm ID; film thickness  = 0.25 µm; Capital Analytical). The GC was programmed from 70°C to 180°C at 30°C/min and then at 5°C/min to 280°C, with a 1-min initial isothermal and a 10-min final isothermal hold. Helium was the carrier gas at a constant flow rate of 1 mL/min. The GC was operated in splitless mode (60 s) at an injector temperature of 250°C. Electron impact ionization (EI) mass spectra were recorded at 70 eV, a source temperature of 230°C, and an interface temperature of 315°C. Data acquisition and storage were performed with the GC-MS software MSD ChemStation for Windows (Agilent Technologies). Peak areas were obtained by manual integration.

3,7-dimethyl-2,6-octadienoic acid (90+% purity) was obtained from Alfa Aesar (Karlsruhe, Germany) and contained two isomers, (*E*)-3,7-dimethyl-2,6-octadienoic acid (geranic acid) and (*Z*)-3,7-dimethyl-2,6-octadienoic acid (nerolic acid). Resacetophenone (99% purity) was purchased from Sigma Aldrich (Steinheim, Germany).

Nerolic acid was identified in the natural samples by comparing the mass spectrum and retention time with data obtained by analysis of the synthetic mixture of isomers of 3,7-dimethyl-2,6-octadienoic acid. Resacetophenone was identified by comparing its mass spectrum and retention time with data obtained by the analysis of the synthetic reference compound. Mellein and its derivatives as well as micromolide and the other γ-lactones were identified by comparing their mass spectra and retention indices with earlier analyses [1]. Hydrocarbons were identified as previously described [76].

### Spatial distribution of antimicrobials on cockroach and cocoon

The aim of this analysis was to determine the absolute and relative amounts of compounds found on the two layers that surround *A. compressa* larvae: the host cockroach and the cocoon. *A. compressa* larvae spin their cocoon inside the empty cockroach carcass. The completed cocoon is firmly attached to the cockroach carcass via innumerous silk threads that strongly adhere to the inner side of the cockroach cuticle. Due to this strong interconnection the cocoon and cockroach cuticle cannot be completely separated from each other for independent chemical analysis.

We therefore established a method to separate cocoons and cockroach carcasses for chemical analyses. When diaphanoscopy indicated that *A. compressa* larvae had eroded the host tissue (8.3±0.5 days after oviposition), the larvae were removed from their hosts and placed in “ersatz hosts” for cocoon spinning (*n* = 31). To this end, parasitized cockroaches were carefully dissected by opening up the abdominal cuticle with sterile dissecting scissors. The larvae were removed and transferred to glass vials (1.5 mL, outer diameter 3.4 mm, height 23.9 mm), which were partly closed with Parafilm “M” to prevent the larvae from escaping but to allow gas exchange (Figure S1). The remaining empty cockroach carcasses were frozen at -20°C immediately after dissection until chemical analyses. The glass vials containing the larvae were incubated at 27°C and the larvae monitored daily. During the next few days 28 of the 31 transferred larvae spun cocoons inside the glass vials (Figure S1). On day 11 after oviposition the cocoons appeared to be completed from the outside. On day 15 after oviposition they were frozen at −20°C until chemical analyses were conducted.

Prior to chemical analysis vials containing cocoons were thawed, the cocoons retrieved from the vials and the larvae carefully removed from the cocoons by opening the cocoons with scissors. For chemical analyses thawed cockroaches and cocoons were singly washed in dichloromethane (DCM; cockroaches: 4 mL; cocoons: 3 mL) containing 0.05 mg mL^−1^ octadecane as an internal standard under gentle agitation for 2 hrs. Because in almost all of the samples some cocoon threads remained in the vials, the vials were also extracted (in 1.5 mL DCM containing 0.05 mg mL^−1^ octadecane) and analyzed. The extracts of cockroaches and cocoons were diluted 1∶2 with DCM. An aliquot of 1 µl of each sample was analyzed by GC/MS.

The peak areas obtained for the cocoons that had been removed from the vials and the peak areas attained for the respective vials were added up to determine the actual total amount of substances the larvae deployed. We included only those peaks in the analyses that occurred in at least 50% of the samples of at least one of the groups (cocoons or cockroaches). Furthermore we excluded peaks that accounted for less than 0.01% on average of the total peak area in both groups.

Quantification of mellein (and its derivatives) and micromolide (and the other γ-lactones) in individual extracts was done by the internal standard method. For this purpose, calibration curves were created by analyses of a dilution series of synthetic mellein and micromolide dissolved in DCM containing the internal standard. Values given are means ± standard deviation.

Prior to the following statistical analyses all hydrocarbons were removed from the data set, as their origin (cockroach or wasp larva) cannot be unambiguously determined and the focus of the analyses was the spatial distribution of the larval antimicrobials. The amounts of larval substances with proposed antimicrobial activity (“larval substances”: compounds 1, 2, 3, 5, 7, 8, 13, 15, 19, 20, 22, 23, 25 in Table 1) were calculated and log_10_-transformed to achieve normal distributions. The total amount of larval substances as well as the amounts of mellein and micromolide were statistically compared between the cockroach and the respective cocoon samples by *t* tests for paired samples.

Finally, the chemical profiles of cocoons and parasitized cockroaches were evaluated by multivariate statistical analyses. The relative peak areas of the peaks representing larval substances were calculated and subjected to statistical analyses. We first visualized differences between cocoons and cockroaches by non-metric multidimensional scaling (*nmMDS*). Subsequently we performed a formal significance test by analysis of similarity (*ANOSIM*). Finally, the chemical compounds primarily responsible for the observed differences between cocoons and cockroaches were determined by similarity percent analysis (*SIMPER*). All three analyses were based on the Bray-Curtis similarity indices [77], [78]. All statistical analyses were performed with the statistics software package PAST (version 2.2, [79]).

### Analyses of parasitized cockroaches of different developmental stages

We analyzed the temporal deployment of the larval antimicrobials during the development of *A. compressa* offspring from egg to adult emergence. As the rate of development differed despite controlled conditions, specimens of the same age varied somewhat in both the stage of larval development and the state of the cockroach. Therefore, we assigned the nests to seven different groups according to the state of the cockroach and larva instead of simply their age and give the average age of the different developmental stages of the nests as “days after oviposition” (Figure S2).

Parasitized cockroaches incubated at 27°C were monitored every day. When the parasitized cockroaches had reached the specified stages (Figure S2; *n* = 9, 10, 11, 10, 10, 9, 10) they were removed from the conditioning cabinet and frozen at −20°C. For chemical analyses, the cockroaches were thawed and individually extracted in 4 mL DCM containing 0.05 mg mL^−1^ octadecane as an internal standard under gentle agitation for 2 hrs. An aliquot of 1 µl of each sample was analyzed by GC/MS.

In this analysis we included the nine compounds (compounds 3, 5, 8, 13, 19, 20, 22, 23, 25 in Table 1 and Figure 1) previously reported from the larval secretion [1]. Quantification was done as described above. The combined amounts of the nine larval substances, as well as the amounts of mellein and micromolide were compared between developmental stages with Kruskal-Wallis-Tests, followed by Mann-Whitney pairwise comparisons with Bonferroni correction. Furthermore, the relationship between the amounts of mellein and micromolide within one sample at the “cocoon” stage was analyzed by a Spearman rank correlation.

### Headspace SPME-GC/MS sampling and analysis

We collected volatile compounds of parasitized cockroaches using headspace solid-phase microextraction (HS-SPME) and analyzed them by GC/MS. Cockroaches (*n* = 6) were taken out of the conditioning cabinet at the ‘cocoon’ stage (about 11 days after oviposition) and individually transferred to 40 mL glass vials which were then closed with lids equipped with PTFE seals. A SPME-fiber (coated with a 100-µm polydimethylsiloxane film; SUPELCO, purchased from Sigma Aldrich, Steinheim, Germany) was inserted through a little hole in the lid into the sample vial headspace at a distance of approximately 2 cm from the cockroach. The fiber was exposed in the sample headspace for 60 min at a constant temperature of 27°C and then immediately thermally desorbed splitless in the GC injection port at 250°C for 5 min. The identity of mellein was verified by comparing mass spectrum and retention time with data obtained for synthetic mellein.

At the end of the bacterial challenge assays (see below) a similar HS-SPME-GC/MS sampling and analysis was performed to control for the presence of mellein in the headspace of the petri dishes containing either parasitized cockroaches or filter papers impregnated with mellein (*n* = 10 each). The SPME-fiber was inserted into each petri dish through a little hole in the lower half of the petri dish (flame cut with a hot dissecting needle) and exposed for 30 min at 23°C. The fiber was immediately analyzed by GC/MS as described above. The median peak areas of mellein obtained from the headspace of cockroaches and filter papers were compared by an exact Mann-Whitney *U* test.

### Microbial challenge assays

#### Bacterial challenge assays

For the tests of antibacterial activity we used the gram-negative bacterium *Serratia marcescens* as target strain, because 1) it is a known entomopathogen [80], [81], [82], 2) we had previously isolated it from *P. americana* cockroaches [1], and 3) we could show that the larval secretion of *A. compressa* as well as synthetic mellein inhibit its growth in broth microdilution assays [1].

Prior to the actual tests, growth curves and calibration curves for the relationship between colony forming units (CFUs)/mL and optical density (OD) were determined for the test organism *S. marcescens*. For the antibacterial assays bacterial suspensions were prepared from cultures grown in lysogeny broth (LB) and harvested in the mid-exponential growth phase. An aliquot of 50 µl of a bacterial suspension containing 1.5×10^6^ CFUs/mL in LB were inoculated on petri dishes containing LB agar. Cubes of the inoculated agar (1×1×0.5 cm) were cut out of the plate with a sterile scalpel and used for the bacterial challenge assays.

For the assays parasitized cockroaches (*n* = 10) were taken out of the conditioning cabinet ten days after oviposition, placed singly in petri dishes (55×15 mm; volume ca. 25 mL) and incubated at 27°C. After 48 hrs, one agar cube freshly inoculated with *S. marcescens* was placed in each petri dish at a distance of approximately 2 cm from the cockroach. As controls, agar cubes obtained from the same inoculated plates were placed in petri dishes without cockroaches. To avoid desiccation of the agar cubes, the closed petri dishes were placed in a plastic box (20×20×6 cm) equipped with an open petri dish filled with sterile water. After incubation at 27°C for 24 hrs the petri dishes were inspected and photographed with a Sony NEX-5 digital camera.

Subsequently, the agar cubes were individually transferred to reaction tubes containing 1 mL of LB broth and vigorously vortexed. Aliquots of the so obtained bacterial suspensions were transferred to a counting chamber (Neubauer improved hemocytometer; Marienfeld, Lauda Königshofen, Germany) and photographed with an Olympus DP20 microscope camera (Olympus Deutschland GmbH, Hamburg, Germany) attached to a Leitz Ortholux II light microscope (Leica AG, Solms, Germany). The number of bacteria in each sample was enumerated on the photos using the image editing program Paint.NET (Version 3.5.10; www.getpaint.net/download.html) and the software Mousometer (Version 3.0; www.mousometer.de/download/). The median numbers of bacteria gained from the test agar cubes (from petri dishes with cockroaches) and control agar cubes (from petri dishes without cockroaches) were compared with an exact Mann-Whitney *U* test.

#### Fungal challenge assays

For the fungal challenge assays we used the two filamentous fungi *A. sydowii* and *A. nomius*, which had been isolated from failed *A. compressa* nests in this study (Text S1), as well as the entomopathogenic fungus *M. brunneum* (strain KVL 03-143/Ma 275; previously referred to as *M. anisopliae*, but now separated as a sister species [3], [83]) as target strains. *M. brunneum* is a well-known entomopathogenic fungus that has been isolated from many different insect species [83].

Fungal spores were harvested from agar plates sustaining fully grown and heavily sporulating fungal colonies. They were suspended in 50 µl Sabouraud-dextrose (SD) broth (concentration *A. sydowii*: 6.4×10^4^ conidia/mL; *A. nomius*: 6.3×10^4^ conidia/mL) or malt extract broth (*M. brunneum*: 5.3×10^4^ spores/mL) and inoculated on petri dishes containing SD or malt extract agar. Cubes of the inoculated agar (1×1×0.5 cm) were cut out of the plate with a sterile scalpel and used for the fungal challenge assays.

Cubes of so seeded agar were placed in petri dishes (55×15 mm) containing parasitized cockroaches or in control dishes without cockroaches (*n* = 5 each) as described above. Petri dishes were incubated at 23°C (*M. brunneum*) or 27°C (*A. sydowii* and *A. nomius*) and checked and photographed every 24 hrs (with a Sony NEX-5 digital camera mounted on a Zeiss OPMI stereomicroscope) for 3 days.

The quantification of fungal growth for *A. sydowii* and *M. brunneum* was conducted using the graphics software ImageJ (1.47v; http://imagej.nih.gov/ij). Photographs taken on day three of the experiment were loaded into the software, first converted to 32bit grayscale and subsequently to indexed color images (lookup table “royal”). Histograms displaying the distributions of pixel values (representing measures of “brightness”; the range of values between the minimum and maximum divided into 256 bins) were produced and the list of values saved. Exploration of the original photographs and the pseudocolor images revealed that the areas of the cubes that were covered with fungus could clearly be distinguished from the areas without fungal growth based on the pixel values. Thus, pixel values of 0 to 99 (from black to dark blue) were defined as “no fungal growth” and pixel values of 100–255 (from light blue to white) as “fungal growth” (see Figure S5). Next, the percentage of the area of the agar cube surface that showed fungal growth was calculated and compared between test cubes (from petri dishes with cockroaches) and controls (from petri dishes without cockroaches) with exact Mann-Whitney *U* tests.

*A. nomius* colonies covered the complete surfaces of the agar cubes and, in contrast to *A. sydowii* and *M. brunneum*, its hyphae protruded considerably from the margins of the agar cubes. The quantification of growth of *A. nomius* was adapted accordingly. Using ImageJ the photographs taken on day three of the experiment were converted to 8bit grayscale and subsequently to binary (black and white) images, so that the fungal mycelia appeared in black. Then the areas of the fungal mycelia were calculated and compared among test and control cubes with an exact Mann-Whitney *U* test.

#### Effect of vaporous mellein on microorganisms

In order to test whether vaporous synthetic (*R*)-(-)-mellein (purchased from Cayman Chemical, Ann Arbor, MI, USA) can inhibit bacterial and fungal growth we repeated the above described experiment for *S. marcescens* (*n* = 10), *A. sydowii* (*n* = 5), and *M. brunneum* (*n* = 5), but used filter papers (1×3 cm) impregnated with mellein (80 µl of a solution containing 23.75 mg mellein mL^-1^ DCM) instead of the parasitized cockroaches. The amount of mellein added to one filter paper was equivalent to the average amount of mellein found on one single parasitized cockroach about ten days after oviposition in a previous study (one “nest equivalent”  = 1.9 mg; [1]). The solvent was allowed to vaporize for 10 min before the filter paper disks were placed in the petri dishes. The control petri dish contained a filter paper soaked with DCM only. Data analyses were conducted as described above.

## Supporting Information

Figure S1 **Photographs of an** ***A. compressa*** **larva and cocoon inside “ersatz host”-glass vials.** (A) *A. compressa* larva transferred from its cockroach host to a glass vial that functions as “ersatz host” after it had eroded the cockroach tissue completely. (B) Cocoon built by an *A. compressa* larva inside the glass vial.
(TIF)

Figure S2 **Illustration of the developmental stages of** ***A. compressa*** **on** ***P. americana*** **.** Illustration of the developmental stages used for the determination of the temporal deployment of antimicrobial substances on *P. americana* cockroaches parasitized by *A. compressa*. (A) Cockroach with egg (yellow arrow) attached to one middle coxa (“egg” stage; 1±0 days after oviposition; *n* = 9), (B) cockroach with big larva (blue arrow) still sitting at the oviposition site (“big larva” stage; 5.8±0.6 days after oviposition; *n* = 10), (C) – (E) cockroaches with larva inside, (C) “thin cockroach” state (7.4±0.5 days after oviposition; *n* = 11), (D) “thick cockroach” state (8.8±0.8 days after oviposition; *n* = 10), (E) diaphanoscopy of a cockroach with a larva (encircled in blue) inside, (F) – (H) cockroaches with cocoon inside, (F) cockroach on first day of visible cocoon inside (“cocoon” stage; 10±0.9 days after oviposition; *n* = 10), (G) cockroach 20 days after oviposition (“20 days” stage; 20±0 days after oviposition; *n* = 9), (H) diaphanoscopy of a cockroach with a cocoon (encircled in white) inside, and (I) cockroach after emergence of the adult wasp (“emergence” stage; 39.4±1.9 days after oviposition; *n* = 10).
(TIF)

Figure S3 **Sequence alignment of red and white** ***Serratia marcescens*** **colonies obtained from control and test agar cubes in the bacterial challenge assays (see main text for details).** The partial 16S rDNA sequences were obtained by Sanger sequencing with oligonucleotide primers fD1 (fwd) and rP2 (rev) (see Supplemental text 1 for further details).
(TIF)

Figure S4 **Estimated concentration of mellein in parasitized cockroaches during the developmental phase of** ***A. compressa*** **.** The amounts of mellein and the mean amount of water present in parasitized cockroaches of the different developmental stages were used to estimate the concentration of mellein in parasitized cockroaches. To this end the amount of water was estimated gravimetrically. Parasitized cockroaches of the developmental stages “thin roach”, “thick roach”, “cocoon”, “20 days” and “emergence” (*n* = 6 per stage) were weighed (Sartorius M-Pact AX124), dried at 50°C and weighed again. The difference between the wet weight and dry weight was taken as an estimate for the amount of water contained in the parasitized cockroaches. As it is not possible to determine the amount of water by gravimetry and the amount of antimicrobials by GC/MS of the same individuals, a new set of parasitized cockroaches was used for gravimetry. The green line shows the IC_50_ value (i.e. the concentration of the antimicrobial compound that is required to inhibit 50% of microbial growth) for mellein against *S. marcescens* determined in a previous study (Herzner et al. 2013).
(TIF)

Figure S5 **Illustration of the method used to estimate fungal growth on agar cubes.** Original photographs of the (A) control and (D) test agar cubes were first converted to 32bit gray scale images (B) and (E) and subsequently to indexed color images (lookup table “royal” in ImageJ) (C) and (F) to accentuate the differences between the clean agar surface and the areas overgrown with fungus. Pixel values (as a measure of “brightness”) from black to dark blue (0–99) were defined as “no fungal growth”, pixel values from light blue to white (100–255) were defined as “fungal growth”. Finally the percent area of the agar cube that was overgrown by fungus was calculated.
(TIF)

Table S1 **Results of the SIMPER analysis.** Compounds are ordered by their individual contributions.
(PDF)

Table S2 **Statistical analyses of the temporal deployment of the larval secretion.** Pairwise comparisons (Mann-Whitney *U* tests) of the median amounts of all larval substances combined found on parasitized cockroaches of different developmental stages. For a detailed description of the stages see Figure S2 and text. The values depict the levels at which the differences are significant (Bonferroni corrected). n.s. =  not significant.
(PDF)

Table S3 **Statistical analyses of the temporal deployment of micromolide and mellein.** Pairwise comparisons (Mann-Whitney *U* tests) of the median amounts of micromolide (upper right) and mellein (lower left) found on parasitized cockroaches of different developmental stages. For a detailed description of the stages see Figure S2 and text. The values depict the levels at which the differences are significant (Bonferroni corrected). n.s. =  not significant.
(PDF)

Text S1 **Methods, Results and Discussion of the molecular genetic analyses used to compare the red and whitish bacterial colonies obtained in the bacterial challenge assays, as well as for the isolation and identification of the** ***Aspergillus*** **fungi.**
(PDF)

## References

[1] Herzner G, Schlecht A, Dollhofer V, Parzefall C, Harrar K, et al (2013) Larvae of the parasitoid wasp *Ampulex compressa* sanitize their host, the American cockroach, with a blend of antimicrobials. P Natl Acad Sci U S A 110: 1369–1374. 10.1073/pnas.1213384110 PMC3557021 23297195

[2] Tragust S, Mitteregger B, Barone V, Konrad M, Ugelvig LV, et al (2013) Ants disinfect fungus-exposed brood by oral uptake and spread fo their poison. Curr Biol 23: 76–82. 23246409 10.1016/j.cub.2012.11.034

[3] Tragust S, Ugelvig LV, Chapuisat M, Heinze J, Cremer S (2013) Pupal cocoons affect sanitary brood care and limit fungal infections in ant colonies. BMC Evol Biol 13: 225. 24125481 10.1186/1471-2148-13-225 PMC3854126

[4] Vilcinskas A, Mukherjee K, Vogel H (2013) Expansion of the antimicrobial peptide repertoire in the invasive ladybird *Harmonia axyridis* . P Royal Soc B: Biol Sci 280: 20122113. 10.1098/rspb.2012.2113 PMC3574431 23173204

[5] Rozen DE, Engelmoer DJP, Smiseth PT (2008) Antimicrobial strategies in burying beetles breeding on carrion. P Natl Acad Sci 105: 17890–17895. 10.1073/pnas.0805403105 PMC2584725 19001269

[6] Gross J, Eben A, Müller I, Wensing A (2010) A well protected intruder: The effective antimicrobial defence of the invasive ladybird *Harmonia axyridis* . J Chem Ecol 36: 1180–1188. 20890794 10.1007/s10886-010-9867-2

[7] Gross J, Schumacher K, Schmidtberg H, Vilcinskas A (2008) Protected by fumigants: beetle perfumes in antimicrobial defense. J Chem Ecol 34: 179–188. 18236110 10.1007/s10886-007-9416-9

[8] Herzner G, Strohm E (2007) Fighting fungi with physics: food wrapping by a solitary wasp prevents water condensation. Curr Biol 17: R46–R47. 17240324 10.1016/j.cub.2006.11.060

[9] Kaltenpoth M, Goettler W, Herzner G, Strohm E (2005) Symbiotic bacteria protect wasp larvae from fungal infestation. Curr Biol 15: 475–479. 15753044 10.1016/j.cub.2004.12.084

[10] Rosengaus RB, Mead K, Du Comb WS, Benson RW, Godoy VG (2013) Nest sanitation through defecation: antifungal properties of wood cockroach feces. Naturwissenschaften 100: 1051–1059. 24271031 10.1007/s00114-013-1110-x

[11] Schmidtberg H, Röhrich C, Vogel H, Vilcinskas A (2013) A switch from constitutive chemical defence to inducible innate immune responses in the invasive ladybird *Harmonia axyridis* . Biol Letters 9: 20130006. 10.1098/rsbl.2013.0006 PMC3645030 23466480

[12] Bulmer MS, Bachelet I, Raman R, Rosengaus RB, Sasisekharan R (2009) Targeting an antimicrobial effector function in insect immunity as a pest control strategy. P Natl Acad Sci U S A 106: 12652–12657. 10.1073/pnas.0904063106 PMC2722268 19506247

[13] Hamilton C, Lay F, Bulmer MS (2011) Subterranean termite prophylactic secretions and external antifungal defenses. J Ins Physiol 57: 1259–1266. 10.1016/j.jinsphys.2011.05.016 21708164

[14] Rolff J, Reynolds SE (2009) Insect Infection and Immunity. Evolution, Ecology and Mechanisms. Oxford: Oxford University Press.

[15] Beckage NE, editor (2008) Insect immunology. San Diego, USA: Academic Press.

[16] Reber A, Purcell J, Buechel SD, Buri P, Chapuisat M (2011) The expression and impact of antifungal grooming in ants. J Evol Biol 24: 954–964. 21306465 10.1111/j.1420-9101.2011.02230.x

[17] Griffiths HM, Hughes WOH (2010) Hitchhiking and the removal of microbial contaminants by the leaf-cutting ant *Atta colombica* . Ecol Entomol 35: 529–537.

[18] Batra S, Bohart GE (1969) Alkali bees: response of adults to pathogenic fungi in brood cells. Science 165: 607–608. 17770861 10.1126/science.165.3893.607

[19] Chouvenc T, Efstathion CA, Elliott ML, Su N-Y (2013) Extended disease resistance emerging from the faecal nest of a subterranean termite. P Royal Soc B: Biol Sci 280: 20131885. 10.1098/rspb.2013.1885 PMC3779336 24048157

[20] Scott JJ, Oh DC, Yuceer MC, Klepzig KD, Clardy J, et al (2008) Bacterial protection of beetle-fungus mutualism. Science 322: 63–63. 18832638 10.1126/science.1160423 PMC2761720

[21] Currie CR, Scott JA, Summerbell RC, Malloch D (1999) Fungus-growing ants use antibiotic-producing bacteria to control garden parasites. Nature 398: 701–704.

[22] Röhrich CR, Ngwa CJ, Wiesner J, Schmidtberg H, Degenkolb T, et al (2012) Harmonine, a defence compound from the harlequin ladybird, inhibits mycobacterial growth and demonstrates multi-stage antimalarial activity. Biol Letters 8: 308–311. 10.1098/rsbl.2011.0760 PMC3297383 21937493

[23] Yek SH, Nash DR, Jensen AB, Boomsma JJ (2012) Regulation and specificity of antifungal metapleural gland secretion in leaf-cutting ants. P Roy Soc B: Biol Sci 279: 4215–4222. 10.1098/rspb.2012.1458 PMC3441083 22915672

[24] Gasch T, Schott M, Wehrenfennig C, Düring R-A, Vilcinskas A (2013) Multifunctional weaponry: The chemical defenses of earwigs. J Ins Physiol 59: 1186–1193. 10.1016/j.jinsphys.2013.09.006 24090659

[25] Kroiss J, Kaltenpoth M, Schneider B, Schwinger MG, Hertweck C, et al (2010) Symbiotic streptomycetes provide antibiotic combination prophylaxis for wasp offspring. Nat Chem Biol 6: 261–263. 20190763 10.1038/nchembio.331

[26] Koehler S, Doubský J, Kaltenpoth M (2013) Dynamics of symbiont-mediated antibiotic production reveal efficient long-term protection for beewolf offspring. Front Zool 10: 3. 23369509 10.1186/1742-9994-10-3 PMC3599432

[27] Keasar T, Sheffer N, Glusman G, Libersat F (2006) Host-handling behavior: An innate component of foraging behavior in the parasitoid wasp *Ampulex compressa* . Ethology 112: 699–706.

[28] Libersat F (2003) Wasp uses venom cocktail to manipulate the behavior of its cockraoch prey. J Comp Physiol A 189: 497–508. 10.1007/s00359-003-0432-0 12898169

[29] Williams FX (1942) *Ampulex compressa* (Fabr.), a cockroach hunting wasp introduced from New Caledonia into Hawaii. P Hawaii Entomol Soc 11: 221–233.

[30] Heitmans WRB (1990) Which information is used to assess host size and sex of the progeny? Experiments with a solitary aculeate ectoparasitoid of cockroaches. Proceedings of the Section Experimental and Applied Entomology of the Netherlands Entomological Society 1: 13–18.

[31] Fakoorziba M, Eghbal F, Hassanzadeh J, Moemenbellah-Fard M (2010) Cockroaches (*Periplaneta americana* and *Blattella germanica*) as potential vectors of the pathogenic bacteria found in nosocomial infections. Ann Trop Med Parasit 104: 521–528. 20863441 10.1179/136485910X12786389891326

[32] Prado AM, Gir E, Pereira MS, Reis C, Pimenta FC (2006) Profile of antimicrobial resistance of bacteria isolated from cockroaches (*Periplaneta americana*) in a Brazilian health care institution. Braz J Infect Dis 10: 26–32. 16767312 10.1590/s1413-86702006000100006

[33] Pai H-H, Chen W-C, Peng C-F (2004) Cockroaches as potential vectors of nosocomial infections. Infect Cont Hosp Ep 25: 979–984. 10.1086/502330 15566034

[34] Pai H-H, Chen W-C, Peng C-F (2005) Isolation of bacteria with antibiotic resistance from household cockroaches (*Periplaneta americana* and *Blattella germanica*). Acta Trop 93: 259–265. 15716054 10.1016/j.actatropica.2004.11.006

[35] Chaichanawongsaroj N, Vanichayatanarak K, Pipatkullachat T, Polrojpanya M, Somkiatcharoen S (2004) Isolation of gram-negative bacteria from cockroaches trapped from urban environment. SE Asian J Trop Med 35: 681–684. 15689087

[36] Baumholtz MA, Parish LC, Witkowski JA, Nutting WB (1997) The medical importance of cockroaches. Int J Dermatol 36: 90–96. 9109002 10.1046/j.1365-4362.1997.00077.x

[37] Trienens M, Keller NP, Rohlfs M (2010) Fruit, flies and filamentous fungi–experimental analysis of animal–microbe competition using *Drosophila melanogaster* and *Aspergillus* mould as a model system. OIKOS 119: 1765–1775.

[38] Janzen D (1977) Why fruits rot, seeds mold, and meat spoils. Am Nat 111: 691–713.

[39] Ma C, Case RJ, Wang Y, Zhang HJ, Tan GT, et al (2005) Anti-tuberculosis constituents from the stem bark of *Micromelum hirsutum* . Planta Med 71: 261–267. 15770548 10.1055/s-2005-837826 PMC2940840

[40] Dai JR, Carté BK, Sidebottom PJ, Yew ALS, Ng SB, et al (2001) Circumdatin G, a new alkaloid from the fungus *Aspergillus ochraceus* . J Nat Prod 64: 125–126. 11170686 10.1021/np000381u

[41] Krohn K, Bahramsari R, Flörke U, Ludewig K, Kliche-Spory C, et al (1997) Dihydroisocoumarins from fungi: Isolation, structure elucidation, circular dichroism and biological activity. Phytochemistry 45: 313–320. 9141717 10.1016/s0031-9422(96)00854-0

[42] Höller U, König GM, Wright AD (1999) Three new metabolites from marine-derived fungi of the genera *Coniothyrium* and *Microsphaeropsis* . J Nat Prod 62: 114–118. 9917295 10.1021/np980341e

[43] Zhao J, Zhang Y, Wang L, Wang J, Zhang C (2012) Bioactive secondary metabolites from *Nigrospora* sp. LLGLM003, an endophytic fungus of the medicinal plant *Moringa oleifera* Lam. World J Microb and Biot 28: 2107–2112. 10.1007/s11274-012-1015-4 22806033

[44] Oliveira CM, Regasini LO, Silva GH, Pfenning LH, Young MCM, et al (2011) Dihydroisocoumarins produced by *Xylaria* sp. and *Penicillium* sp., endophytic fungi associated with *Alibertia macrophylla* and *Piper aduncum* . Phytochem Lett 4: 93–96.

[45] Feng Z, Nenkep V, Yun K, Zhang D, Choi H, et al (2010) Biotransformation of Bioactive (-)-Mellein by a Marine Isolate of Bacterium *Stappia* sp. J Microbiol Biotechn 20: 985–987. 10.4014/jmb.1002.02012 20622496

[46] El-Mehalawy AA, Abd-Allah NA, Mohammed RM, Abu-Shady MR (2005) Actinomycetes antagonizing plant and human pathogenic fungi, II. Factors affecting antifungal production and chemical characterization of the active components. Int J Agricul Biol 7: 188–196.

[47] Yuan H, He R, Wan B, Wang Y, Pauli GF, et al (2008) Modification of the side chain of micromolide, an anti-tuberculosis natural product. Bioorg Med Chem Lett 18: 5311–5315. 18774716 10.1016/j.bmcl.2008.08.027

[48] Rukachaisirikul V, Arunpanichlert J, Sukpondma Y, Phongpaichit S, Sakayaroj J (2009) Metabolites from the endophytic fungi *Botryosphaeria rhodina* PSU-M35 and PSU-M114. Tetrahedron 65: 10590–10595.

[49] Haak U, Hölldobler B, Bestmann H, Kern F (1996) Species-specificity in trail pheromones and Dufour's gland contents of *Camponotus atriceps* and *C. floridanus* (Hymenoptera: Formicidae). Chemoecology 7: 85–93.

[50] Muneer M, Qamar M, Bahnemann D (2005) Photoinduced electron transfer reaction of few selected organic systems in presence of titanium dioxide. J Mol Catal A: Chemical 234: 151–157.

[51] Lemos AA, Lemos JA, Prado MA, Pimenta FC, Gir E, et al (2006) Cockroaches as carriers of fungi of medical importance. Mycoses 49: 23–25. 10.1111/j.1439-0507.2005.01179.x 16367814

[52] Führer E, Willers D (1986) The anal secretion of the endoparasitic larva *Pimpla turionellae*: sites of production and effects. J Insect Physiol 32: 361–367.

[53] Herzner G, Engl T, Strohm E (2011) Cryptic combat against competing microbes is a costly component of parental care in a digger wasp. Anim Behav 82: 321–328.

[54] Cotter SC, Topham E, Price AJP, Kilner RM (2010) Fitness costs associated with mounting a social immune response. Ecol Lett 13: 1114–1123. 20545735 10.1111/j.1461-0248.2010.01500.x

[55] Degenkolb T, Düring RA, Vilcinskas A (2011) Secondary metabolites released by the burying beetle *Nicrophorus vespilloides*: chemical analyses and possible ecological functions. J Chem Ecol 37: 724–735. 21667150 10.1007/s10886-011-9978-4

[56] Führer E, Rosner S, Schmied A, Wegensteiner R (2001) Studies on the significance of pathogenic fungi in the population dynamics of the lesser spruce sawfly, *Pristiphora abietina* Christ. (Hym., Tenthredinidae). J Applied Entomol 125: 235–242.

[57] Thompson P, Hepburn H (1978) Changes in chemical and mechanical properties of honeybee (*Apis mellifera adansonii* L.) cuticle during development. J Comp Physiol 126: 257–262.

[58] Vänninen I, Hokkanen H (1997) Efficacy of entomopathogenic fungi and nematodes against *Argyresthia conjugella* (Lep.: Yponomeutidae). Entomophaga 42: 377–385.

[59] Gochnauer T, Boch R, Margetts V (1979) Inhibition of *Ascosphaera apis* by citral and geraniol. J Invertebr Pathol 34: 57–61.

[60] Wang L, Dong JY, Song HC, Shen KZ, Wang LM, et al (2008) Screening and isolation of antibacterial activities of the fermentative extracts of freshwater fungi from Yunnan Province, China. Ann Microbiol 58: 579–584.

[61] Sun CM, Toia RF (1993) Biosynthetic studies on ant metabolites of mellein and 2, 4-dihydroxyacetophenone from {1, 2-^13^C_2_} Acetate. J Nat Prod 56: 953–956.

[62] Schulz B, Sucker J, Aust H, Krohn K, Ludewig K, et al (1995) Biologically active secondary metabolites of endophytic *Pezicula* species. Mycol Res 99: 1007–1015.

[63] Hejazi A, Falkiner FR (1997) *Serratia marcescens* . J Med Microbiol 46: 903–912. 9368530 10.1099/00222615-46-11-903

[64] Anğ-Küçüker M, Büyükbaba-Boral Ö, Tolun V, Törümküney D, Susever S, et al (1999) Effect of Some Antibiotics on Pigmentation in *Serratia marcescens* . ZBL Bakt 289: 781–785. 10.1016/s0934-8840(00)80001-8 10705609

[65] Iranshahi M, Shahverdi AR, Mirjani R, Amin G, Shafiee A (2004) Umbelliprenin from *Ferula persica* roots inhibits the red pigment production in *Serratia marcescens* . Z Naturforsch 59c: 506–508. 10.1515/znc-2004-7-809 15813369

[66] Cardoza YJ, Klepzig KD, Raffa KF (2006) Bacteria in oral secretions of an endophytic insect inhibit antagonistic fungi. Ecol Entomol 31: 636–645.

[67] Chen J, Henderson G, Grimm C, Lloyd S, Laine R (1998) Termites fumigate their nests with naphthalene. Nature 392: 558–559.

[68] Chen J, Henderson G, Grimm CC, Lloyd SW, Laine RA (1998) Naphthalene in Formosan subterranean termite carton nests. J Agr Food Chem 46: 2337–2339.

[69] Wiltz BA, Henderson G, Chen J (1998) Effect of naphthalene, butylated hydroxytoluene, dioctyl phthalate, and adipic dioctyl ester, chemicals found in the nests of the formosan subterranean termite (Isoptera: Rhinotermitidae) on a saprophytic *Mucor* sp.(Zygomycetes: Mucorales). Environ Entomol 27: 936–940.

[70] Wright MS, Lax AR, Henderson G, Chen J (2000) Growth response of *Metarhizium anisopliae* to two Formosan subterranean termite nest volatiles, naphthalene and fenchone. Mycologia 92: 42–45.

[71] Rosengaus R, Traniello J, Lefebvre M, Maxmen A (2004) Fungistatic activity of the sternal gland secretion of the dampwood termite *Zootermopsis angusticollis* . Insect Soc 51: 259–264.

[72] Rosengaus RB, Lefebvre ML, Traniello JFA (2000) Inhibition of fungal spore germination by *Nasutitermes*: evidence for a possible antiseptic role of soldier defensive secretions. J Chem Ecol 26: 21–39.

[73] Gross J, Schmidtberg H (2009) Glands of leaf beetle larvae - protective structures against attacking predators and pathogens. In: Jolivet P, Santiago-Blay J, Schmidt M, editors. Research on Chrysomelidae. Leiden, The Netherlands: Koninklijke Brill.pp. 177–189.

[74] Suwannarach N, Kumla J, Bussaban B, Nuangmek W, Matsui K, et al (2013) Biofumigation with the endophytic fungus *Nodulisporium* spp. CMU-UPE34 to control postharvest decay of citrus fruit. Crop Prot 45: 63–70.

[75] Tunc S, Chollet E, Chalier P, Preziosi-Belloy L, Gontard N (2007) Combined effect of volatile antimicrobial agents on the growth of *Penicillium notatum* . Int J Food Microbiol 113: 263–270. 17011661 10.1016/j.ijfoodmicro.2006.07.004

[76] Herzner G, Ruther J, Goller S, Schulz S, Goettler W, et al (2011) Structure, chemical composition and putative function of the postpharyngeal gland of the emerald cockroach wasp, *Ampulex compressa* (Hymenoptera, Ampulicidae). Zoology 114: 36–45. 21256725 10.1016/j.zool.2010.10.002

[77] Clarke K, Green R (1988) Statistical design and analysis for a ‘biological effects’ study. Mar Ecol - Prog Ser 46: 213–226.

[78] Legendre P, Legendre L (1998) Numerical Ecology. Amsterdam: Elsevier Science B.V.

[79] Hammer Ø, Harper DAT, Ryan PD (2001) PAST: Paleontological statistics software package for education and data analysis. Paleo Electronica 4: Art 4: 9pp.

[80] Sikorowski P, Lawrence A, Inglis G (2001) Effects of *Serratia marcescens* on rearing of the tobacco budworm. American Entomologist 47: 51–60.

[81] Li B, Yu R, Liu B, Tang Q, Zhang G, et al (2011) Characterization and comparison of *Serratia marcescens* isolated from edible cactus and from silkworm for virulence potential and chitosan susceptibility. Braz J Microbiol 42: 96–104. 24031610 10.1590/S1517-83822011000100013 PMC3768933

[82] Secil ES, Sevim A, Demirbag Z, Demir I (2012) Isolation, characterization and virulence of bacteria from *Ostrinia nubilalis* (Lepidoptera: Pyralidae). Biologia 67: 767–776.

[83] Bischoff JF, Rehner SA, Humber RA (2009) A multilocus phylogeny of the *Metarhizium anisopliae* lineage. Mycologia 101: 512–530. 19623931 10.3852/07-202

