# Supplementary figures and images for: Multifaceted Defense against Antagonistic Microbes in Developing Offspring of the Parasitoid Wasp Ampulex compressa (Hymenoptera, Ampulicidae)
Source: PLoS One. 2014 Jun 2;9(6):e98784. doi: 10.1371/journal.pone.0098784 (PMC4041758; doi:10.1371/journal.pone.0098784)

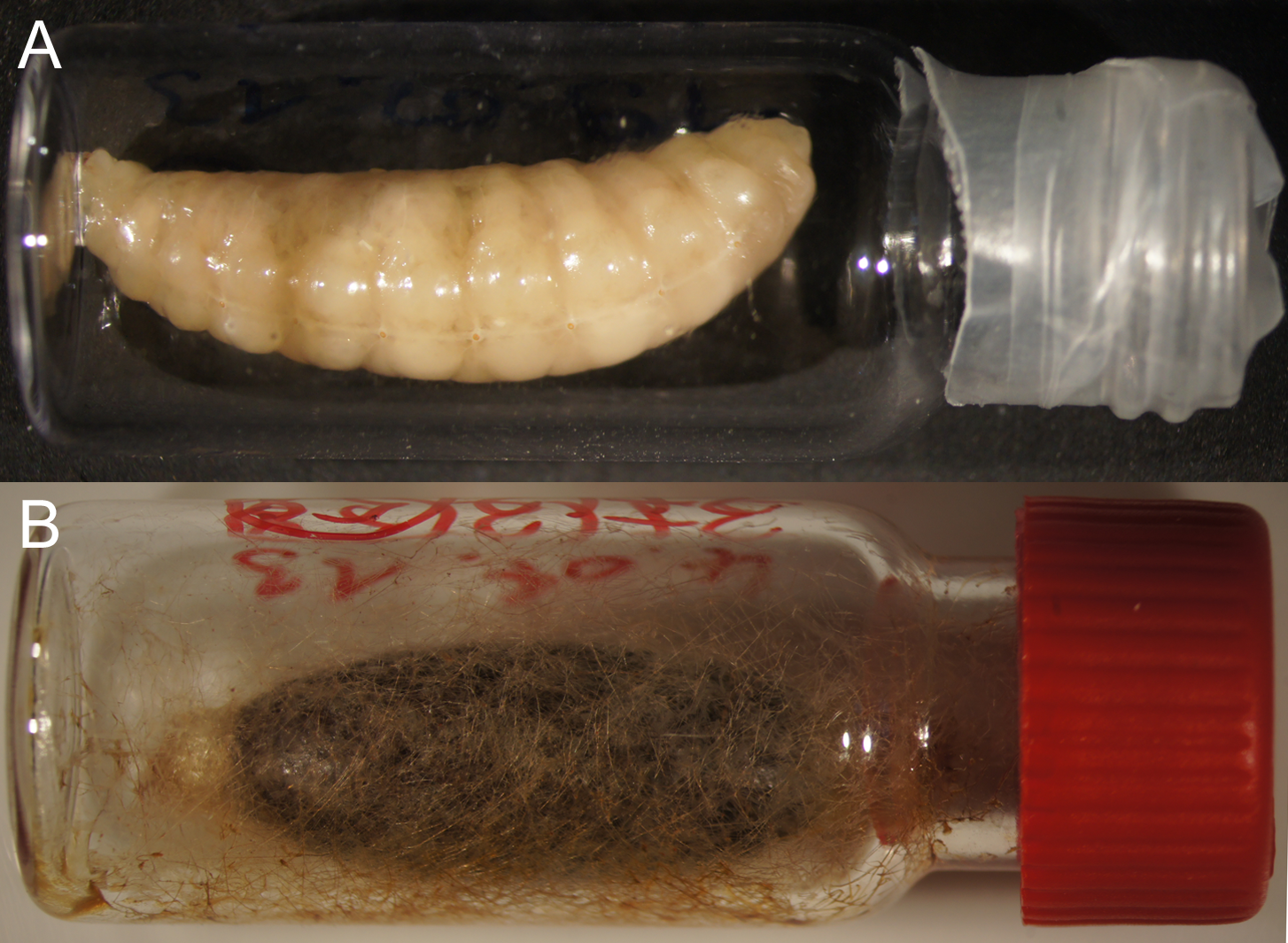

Supplement: Figure S1 — Photographs of an A. compressa larva and cocoon inside “ersatz host”-glass vials. (A) A. compressa larva transferred from its cockroach host to a glass vial that functions as “ersatz host” after it had eroded the cockroach tissue completely. (B) Cocoon built by an A. compressa larva inside the glass vial. (TIF) [file pone.0098784.s001.tif]

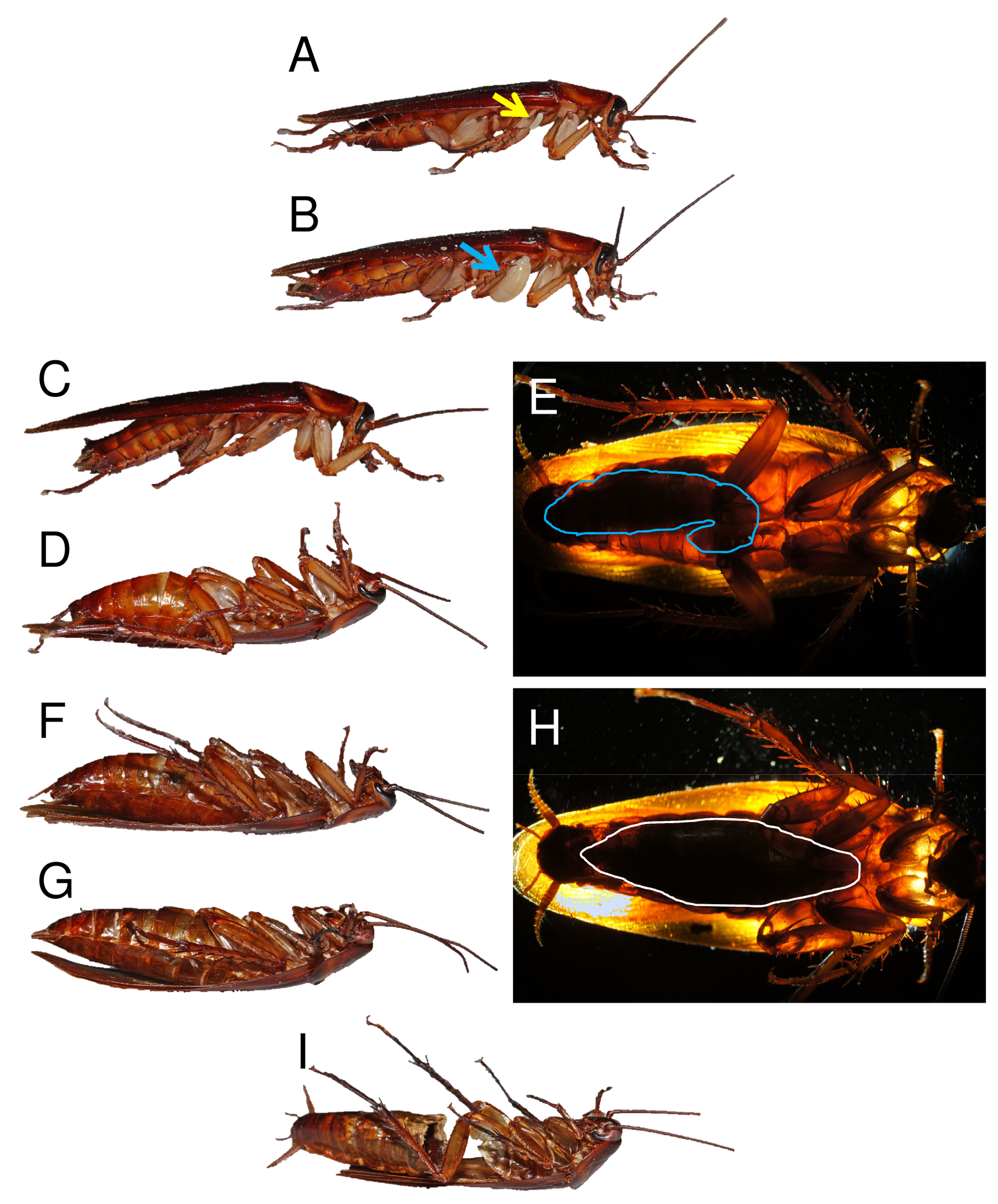

Supplement: Figure S2 — Illustration of the developmental stages of A. compressa on P. americana . Illustration of the developmental stages used for the determination of the temporal deployment of antimicrobial substances on P. americana cockroaches parasitized by A. compressa. (A) Cockroach with egg (yellow arrow) attached to one middle coxa (“egg” stage; 1±0 days after oviposition; n = 9), (B) cockroach with big larva (blue arrow) still sitting at the oviposition site (“big larva” stage; 5.8±0.6 days after oviposition; n = 10), (C) – (E) cockroaches with larva inside, (C) “thin cockroach” state (7.4±0.5 days after oviposition; n = 11), (D) “thick cockroach” state (8.8±0.8 days after oviposition; n = 10), (E) diaphanoscopy of a cockroach with a larva (encircled in blue) inside, (F) – (H) cockroaches with cocoon inside, (F) cockroach on first day of visible cocoon inside (“cocoon” stage; 10±0.9 days after oviposition; n = 10), (G) cockroach 20 days after oviposition (“20 days” stage; 20±0 days after oviposition; n = 9), (H) diaphanoscopy of a cockroach with a cocoon (encircled in white) inside, and (I) cockroach after emergence of the adult wasp (“emergence” stage; 39.4±1.9 days after oviposition; n = 10). (TIF) [file pone.0098784.s002.tif]

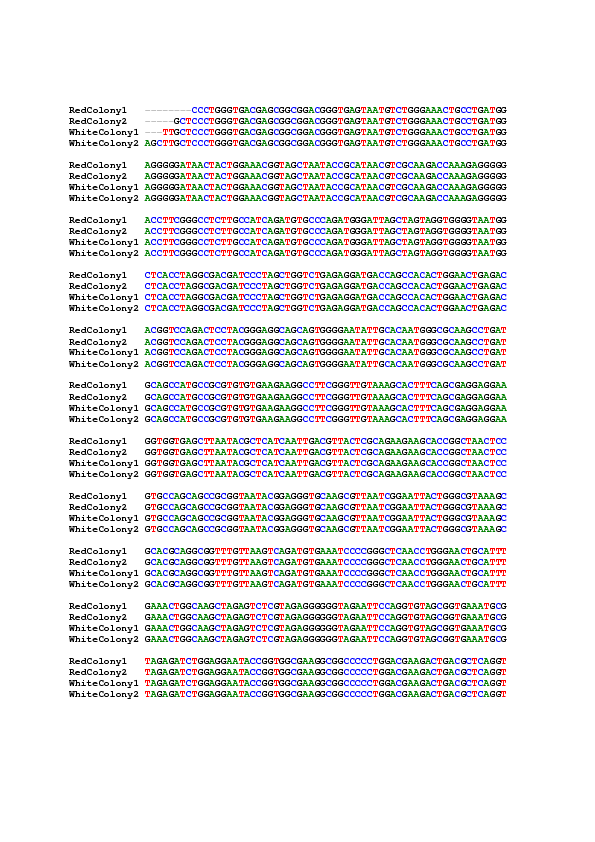

Supplement: Figure S3 — Sequence alignment of red and white Serratia marcescens colonies obtained from control and test agar cubes in the bacterial challenge assays (see main text for details). The partial 16S rDNA sequences were obtained by Sanger sequencing with oligonucleotide primers fD1 (fwd) and rP2 (rev) (see Supplemental text 1 for further details). (TIF) [file pone.0098784.s003.tif]

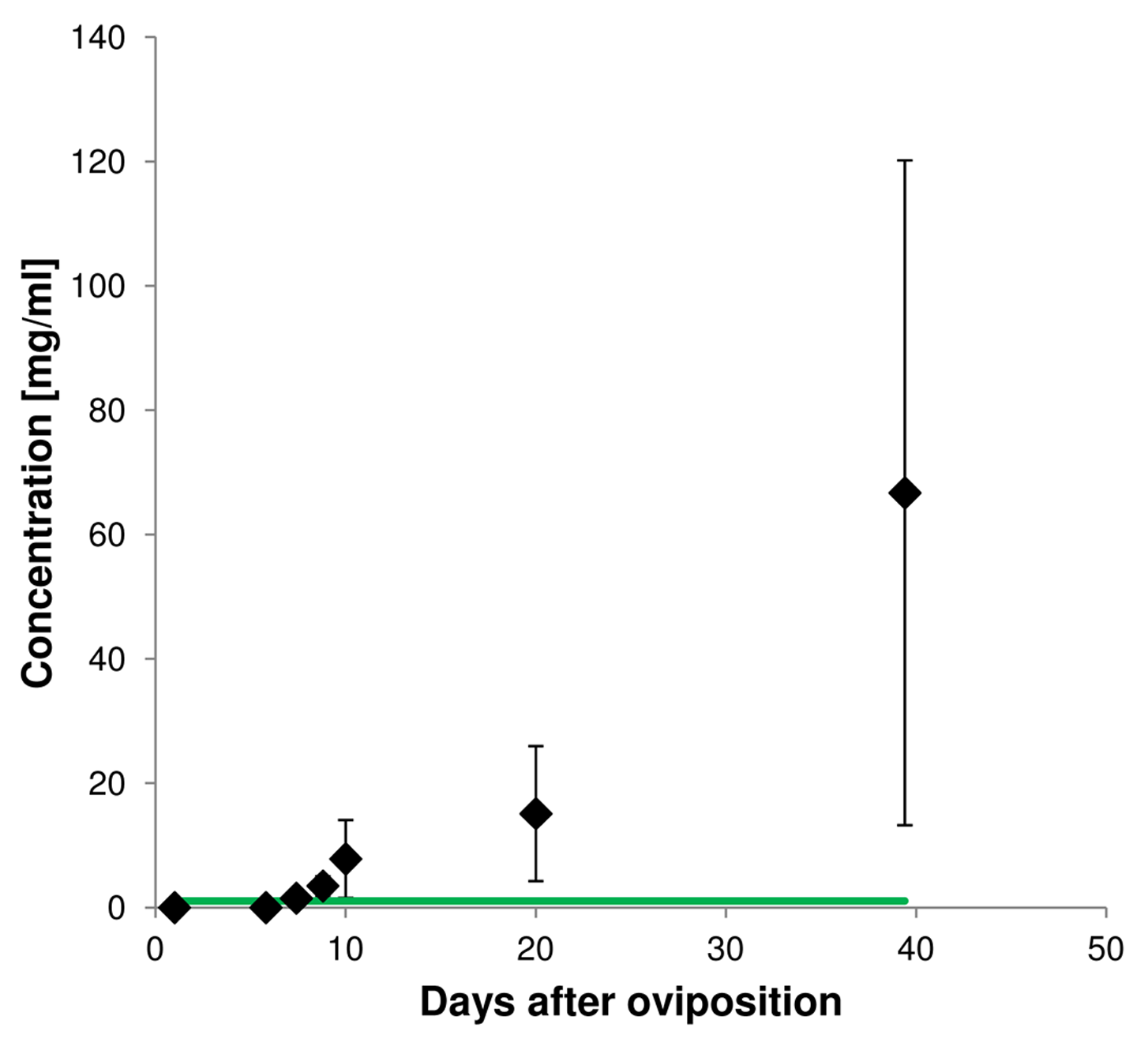

Supplement: Figure S4 — Estimated concentration of mellein in parasitized cockroaches during the developmental phase of A. compressa . The amounts of mellein and the mean amount of water present in parasitized cockroaches of the different developmental stages were used to estimate the concentration of mellein in parasitized cockroaches. To this end the amount of water was estimated gravimetrically. Parasitized cockroaches of the developmental stages “thin roach”, “thick roach”, “cocoon”, “20 days” and “emergence” (n = 6 per stage) were weighed (Sartorius M-Pact AX124), dried at 50°C and weighed again. The difference between the wet weight and dry weight was taken as an estimate for the amount of water contained in the parasitized cockroaches. As it is not possible to determine the amount of water by gravimetry and the amount of antimicrobials by GC/MS of the same individuals, a new set of parasitized cockroaches was used for gravimetry. The green line shows the IC50 value (i.e. the concentration of the antimicrobial compound that is required to inhibit 50% of microbial growth) for mellein against S. marcescens determined in a previous study (Herzner et al. 2013). (TIF) [file pone.0098784.s004.tif]

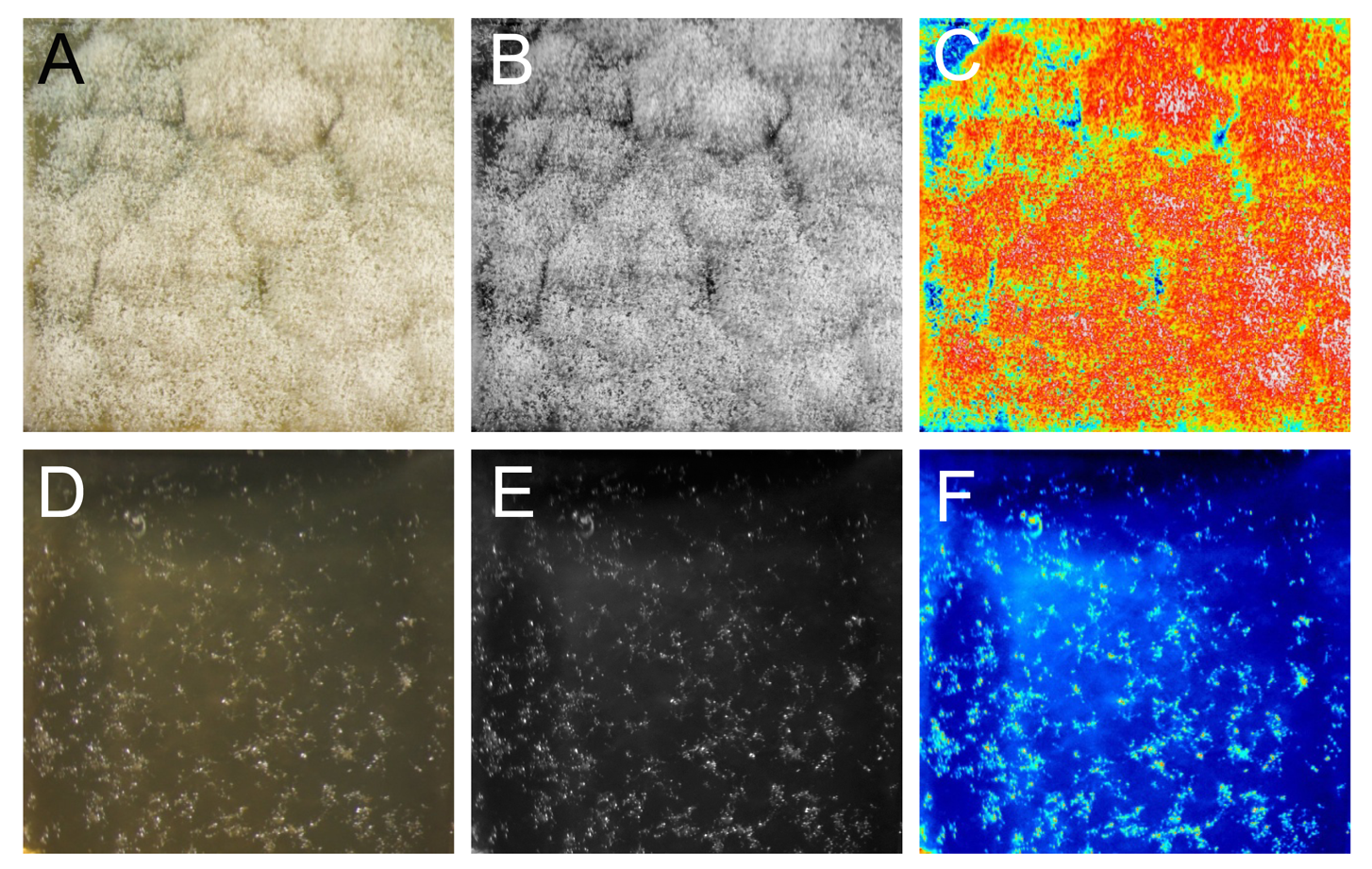

Supplement: Figure S5 — Illustration of the method used to estimate fungal growth on agar cubes. Original photographs of the (A) control and (D) test agar cubes were first converted to 32bit gray scale images (B) and (E) and subsequently to indexed color images (lookup table “royal” in ImageJ) (C) and (F) to accentuate the differences between the clean agar surface and the areas overgrown with fungus. Pixel values (as a measure of “brightness”) from black to dark blue (0–99) were defined as “no fungal growth”, pixel values from light blue to white (100–255) were defined as “fungal growth”. Finally the percent area of the agar cube that was overgrown by fungus was calculated. (TIF) [file pone.0098784.s005.tif]
