# Supplementary material for: Multifaceted Defense against Antagonistic Microbes in Developing Offspring of the Parasitoid Wasp Ampulex compressa (Hymenoptera, Ampulicidae)
Source: PLoS One. 2014 Jun 2;9(6):e98784. doi: 10.1371/journal.pone.0098784 (PMC4041758; doi:10.1371/journal.pone.0098784)
Supplement: Table S1 — Results of the SIMPER analysis. Compounds are ordered by their individual contributions. (PDF) [file pone.0098784.s006.pdf]

Table S1: Results of the SIMPER analysis. Compounds are ordered by their individual contributions.

**Compound**	**Average dissimilarity**	**Individual Contribution [%]**	**Cumulative contribution [%]**
Micromolide	9.11	41.90	41.90
(*R*)-(-)-Mellein	5.45	25.06	66.96
7-Hydroxymellein	4.75	21.87	88.83
(*R*)-Octadecan-4-olide	1.15	5.29	94.12
4-Hydroxymellein	0.40	1.83	95.94
5-Hydroxymellein	0.34	1.58	97.52
Octadeca-9,12-dien-4-olide	0.23	1.05	98.57
(*R*)-Hexadecan-4-olide	0.18	0.82	99.39
Neric acid	0.05	0.21	99.60
Reacetophenone	0.03	0.16	99.76
Unidentified1	0.02	0.11	99.87
Unidentified2	0.01	0.07	99.94
Heptadecan-4-olide	0.01	0.06	100
