# Supplementary material for: Multifaceted Defense against Antagonistic Microbes in Developing Offspring of the Parasitoid Wasp Ampulex compressa (Hymenoptera, Ampulicidae)
Source: PLoS One. 2014 Jun 2;9(6):e98784. doi: 10.1371/journal.pone.0098784 (PMC4041758; doi:10.1371/journal.pone.0098784)
Supplement: Table S2 — Statistical analyses of the temporal deployment of the larval secretion. Pairwise comparisons (Mann-Whitney U tests) of the median amounts of all larval substances combined found on parasitized cockroaches of different developmental stages. For a detailed description of the stages see Figure S2 and text. The values depict the levels at which the differences are significant (Bonferroni corrected). n.s. = not significant. (PDF) [file pone.0098784.s007.pdf]

Table S2. Statistical analyses of the temporal deployment of the larval secretion.

Stage	Egg	Big larva	Thin roach	Thick roach	Cocoon	20 Days	Emergence
Egg		n.s	0.01	0.01	0.01	0.01	0.01
Big larva			0.01	0.01	0.01	0.01	0.01
Thin roach				n.s	0.05	n.s	n.s
Thick roach					n.s	n.s.	n.s
Cocoon						n.s	n.s
20 Days							n.s
Emergence							

Pairwise comparisons (Mann-Whitney *U* tests) of the median amounts of all larval substances combined found on parasitized cockroaches of different developmental stages. For a detailed description of the stages see Figure S2 and text. The values depict the levels at which the differences are significant (Bonferroni corrected).

n.s.= not significant.
