# Supplementary material for: Multifaceted Defense against Antagonistic Microbes in Developing Offspring of the Parasitoid Wasp Ampulex compressa (Hymenoptera, Ampulicidae)
Source: PLoS One. 2014 Jun 2;9(6):e98784. doi: 10.1371/journal.pone.0098784 (PMC4041758; doi:10.1371/journal.pone.0098784)
Supplement: Text S1 — Methods, Results and Discussion of the molecular genetic analyses used to compare the red and whitish bacterial colonies obtained in the bacterial challenge assays, as well as for the isolation and identification of the Aspergillus fungi. (PDF) [file pone.0098784.s009.pdf]

Text S1

Confirmation of the whitish bacterial colonies as *S. marcescens*

Methods

To make sure that the whitish colonies growing on agar cubes incubated in the headspace of parasitized cockroaches were *S. marcescens* and not contaminations, we compared a partial 16S rDNA sequence of these colonies with the red colonies growing on control agar cubes. We set up an additional bacterial challenge assay as described in the Methods section to obtain bacterial colonies for genetic analyses. For genetic analyses, samples of single colonies from two control and two test agar cubes were picked with sterile toothpicks.

The DNA was extracted with a MasterPure™ Complete DNA and RNA Purification Kit (Epicentre Biotechnologies) following the manufacturer's instructions. The DNA was then used as a template for polymerase chain reaction (PCR) with oligonucleotide primers fD1 (forward) and rP2 (reverse) [1] for the amplification of fragments of the 16S rDNA.

PCR reactions were performed on a Biometra T-Gradient Thermocycler in total reaction volumes of 12.5µl containing 1µl of template, 1 x PCR buffer [50mM Tris-HCl pH 9.1, 14mM $(NH_4)SO_4$], 2.5mM $MgCl_2$, 240µM dNTPs, 10pmol of each primer, and 0.5 U of Taq DNA polymerase (Peqlab), respectively. Cycle parameters were as follows: 3min at 95°C, followed by 39 cycles of 95°C for 1min., an annealing temperature of 58°C for 1min, and 72°C for 1min, and a final extension time of 3min at 72 °C.

Two amplicons of each group (red and white bacterial colonies) were purified (PeqGOLD MicroSpin cycle-Pure kit, Peqlab, Germany) and subsequently commercially sequenced (Eurofins MWG Operon, Ebersberg, Germany) with primers fD1/rP2. The obtained 16S rDNA sequences were aligned and compared using the software BioEdit v7.2.3 (Ibis Biosciences, Carlsbad, CA, USA).

Results

The partial 16S rDNA sequences that we obtained for the red (808 and 810bp) and the whitish (813 and 816bp) bacterial colonies in the bacterial challenge assays were identical (Figure S3). Furthermore the sequence showed 100% homology with the *S. marcescens* strain originally isolated from cockroaches in a previous study [2] (GenBank accession number: JX448402) and inoculated on the agar cubes here.

Discussion

The bacteria forming whitish colonies on the agar cubes incubated in the headspace of parasitized cockroaches were genetically identical to the *S. marcescens* strain forming red colonies on the control agar cubes, and do thus represent decolorized colonies of the originally inoculated bacterium.

Isolation and identification of fungi from cockroaches

Methods

To obtain possible antagonistic fungi that might threaten *A. compressa* offspring during their development we probed incubated nests that failed to produce adult *A. compressa* wasps and that appeared to be infested by fungi (occurrence of fungal hyphae and/or conidiophores). Probes of fungi were isolated from four infested, parasitized cockroaches by use of sterile forceps. The fungi were inoculated onto Sabouraud-dextrose (SD) agar plates and placed in a conditioning cabinet at 30°C until visible growth of microbes. For the identification of the fungi, single mature conidiophores were seeded on new SD agar plates at least twice to obtain pure strains. From these colonies samples were taken for identification by Sanger sequencing of rDNA internal transcribed spacer regions (ITS) 1 and

2. In addition, morphological characteristics of the cultivated strains were investigated and recorded.

Growing mycelia of the fungi were sampled, ground in liquid nitrogen and the DNA extracted as described above for *S. marcescens*. The DNA was then used as a template for PCR with oligonucleotide primers ITS1 (forward) and ITS4 (reverse) [3] for the amplification of fragments of rDNA regions ITS1 and ITS2.

PCR reactions and amplicon purification were performed as described above for *S. marcescens*. The amplicons were subsequently sequenced commercially (Eurofins MWG Operon) with primers ITS1/ITS4. The obtained partial ITS sequences were submitted to the international nucleotide sequence library GenBank (see results for accession numbers) and compared to sequences published in GenBank using the BLAStn algorithm of the National Center of Biotechnology Information (NCBI).

Results

From the infested cockroaches we obtained four fungal isolates. Two of the isolates grew rapidly and exhibited intense yellow-green colonies after four days of incubation. One of these isolates was used for genetic analyses. The partial ITS sequence of this fungal isolate (530bp, GenBank accession number: KF978105]) showed 100% homology with *Aspergillus nomius* Kurtzman, Horn and Hesseltine ITS sequences (e.g. GenBank accession number: JF824686.1) in the GenBank database. The two other fungal isolates grew more slowly and developed colonies that were variable in color ranging from beige-brown to dark green after five days. The partial ITS sequence of this fungus (497bp, GenBank accession number: KF978104) had 100% homology with an ITS sequence published for *Aspergillus sydowii* Thom and Church (GenBank accession number: KC253961.1).

Discussion

In this study we isolated and identified the two filamentous fungi *A. nomius* and *A. sydowii* from failed *A. compressa* nests. The ubiquitous fungus *A. nomius* belongs to the section Flavi of the genus *Aspergillus* and is a highly aggressive, fast growing, and aflatoxin-producing species. It is known as an opportunistic facultative entomopathogen, and has also been reported as soil fungus, as mold on stored food and as pathogen in humans [4-8]. *A. sydowii* belongs to the *Aspergillus versicolor* section and has been isolated from insects [9], soil and air, as well as water [10]. It is known to infest grain cultures [11] and to cause Aspergillosis in various animals from corals to humans [12, 13].

Supporting References

1. Weisburg WG, Barns SM, Pelletier DA, Lane DJ (1991) 16S ribosomal DNA amplification for phylogenetic study. J Bacteriol 173: 697-703.
2. Herzner G, Schlecht A, Dollhofer V, Parzefall C, Harrar K, et al. (2013) Larvae of the parasitoid wasp *Ampulex compressa* sanitize their host, the American cockroach, with a blend of antimicrobials. Proc Natl Acad Sci of U S A 110: 1369-1374.
3. White T, Bruns T, Lee S, Taylor J (1990) Amplification and direct sequencing of fungal ribosomal RNA genes for phylogenies. In: Innis M, Gelfand D, Sninsky J, White T, editors. PCR–Protocols: A Guide to Methods and Applications. New York: Academic Press, Inc. pp. 315–322.
4. Kurtzman CP, Horn BW, Hesseltine CW (1987) *Aspergillus nomius*, a new aflatoxin-producing species related to *Aspergillus flavus* and *Aspergillus tamarii*. Anton Leeuw 53: 147-158.
5. Poulsen M, Hughes WOH, Boomsma JJ (2006) Differential resistance and the importance of antibiotic production in *Acromyrmex echinator* leaf-cutting ant castes towards the entomopathogenic fungus *Aspergillus nomius*. Insect Soc 53: 349-355.
6. Olsen M, Johnsson P, Möller T, Paladino R, Lindblad M (2008) *Aspergillus nomius*, an important aflatoxin producer in Brazil nuts? World Mycotoxin Journal 1: 123-126.
7. Manikandan P, Varga J, Kocsubé S, Samson RA, Anita R, et al. (2009) Mycotic keratitis due to *Aspergillus nomius*. J Clin Microbiol 47: 3382-3385.
8. Chouvenc T, Efstathion CA, Elliott ML, Su N-Y (2012) Resource competition between two fungal parasites in subterranean termites. Naturwissenschaften 99: 949-958.
9. Pereira ES, Sarquis MIM, Ferreira-Keppler RL, Hamada N, Alencar YB (2009) Filamentous fungi associated with mosquito larvae (Diptera: Culicidae) in municipalities of the Brazilian Amazon. Neotrop Entomol 38: 352-359.
10. Klich MA (2002) Biogeography of *Aspergillus* species in soil and litter. Mycologia 94: 21-27.
11. Steiner S, Erdmann D, Steidle JLM, Ruther J (2007) Host habitat assessment by a parasitoid using fungal volatiles. Front Zool 4.
12. Alker AP, Smith GW, Kim K (2001) Characterization of *Aspergillus sydowii* (Thom et Church), a fungal pahtogen of Caribbean sea fan corals. Hydrobiologia 460: 105-111.

13. Marfenina OE, Fomicheva GM, Vasilenko OV, Naumova EM, Kul'ko AB (2010) Sporulation in saprophytic and clinical strains of *Aspergillus sydowii* (Bain. & Sart.) Thom & Church under various environmental conditions. Microbiology 2010: 6.
